# The Influence of Carbon Fiber Content and Strain Rate on the Mechanical Properties and Microscopic Damage Evolution of Recycled Aggregate Concrete

**DOI:** 10.3390/ma19183867

**Published:** 2026-09-11

**Authors:** Chenyang Yuan, Jingyu Qi, Yunfei Xie, Weifeng Bai, Junfeng Guan, Jing Liu, Kai Wang, Lielie Li

**Affiliations:** 1 School of Water Conservancy, North China University of Water Resources and Electric Power, Zhengzhou 450046, China; yuanchenyang@ncwu.edu.cn (C.Y.); junfengguan@ncwu.edu.cn (J.G.); 2 Heilongjiang Sanjiang Water Conservancy Construction Management Co., Ltd., Harbin 150090, China; 18342888969@163.com; 3 School of Human Settlements, North China University of Water Resources and Electric Power, Zhengzhou 450046, China; 4 Yantai Laolan Reservoir Resettlement Service Center, Yantai 264003, China; liujsmile@126.com; 5 Yantai Architectural Design and Research Co., Ltd., Yantai 264003, China; ytsywangkai@163.com; 6 School of Civil Engineering and Communication, North China University of Water Resources and Electric Power, Zhengzhou 450045, China; lilielie@ncwu.edu.cn

**Keywords:** carbon fiber, recycled concrete, strain rate, statistical damage theory

## Abstract

This study systematically investigated the effects of carbon fiber (CF) content (0%, 0.15%, 0.3%) and strain rate (10^−5^/s, 10^−4^/s, 10^−3^/s, 10^−2^/s) on the mechanical properties, microstructure, and microscopic damage evolution of carbon fiber-modified recycled concrete (CFRRAC) using uniaxial compression testing, scanning electron microscopy (SEM) observation, acoustic emission (AE), and statistical damage theory. The results indicate that the moderate addition of CF can effectively improve the compactness of the microstructure of the specimen, enhance the strain rate effect of CFRRAC, and improve its initial macroscopic mechanical properties. The microstructure characteristics of specimens with different CF contents and the Stefan effect related to strain rate further affect the initiation and propagation morphology, propagation path, and adjustment process of effective stress skeleton of microcracks during uniaxial compression, leading to regular changes in characteristic parameters characterizing microfracture and yield damage evolution with CF content and strain rate. The above factors collectively determine the evolution characteristics of the macroscopic nonlinear stress–strain behavior of CFRRAC, combined with the CF bridging toughening effect, ultimately resulting in an increase in strength with increasing strain rate and maintaining good ductility. Compared with the specimens without CF doping, the peak stress of CFRRAC increased by 37.16% to 41.18% and the peak strain increased by 22.94% to 36.57% in the strain rate range of 10^−5^ to 10^−2^/s at a dosage of 0.3%. Taking the CFRRAC specimen with a content of 0.3% as an example, compared with the strain rate of 10^−5^/s, the peak stress of the specimen increased by 9.31%, 18.66%, and 31.24% at strain rates ranging from 10^−4^ to 10^−2^/s, respectively. The research results can provide theoretical support for the promotion and application of CFRRAC in the engineering field.

## 1. Introduction

As a primary structural material, concrete plays an irreplaceable role in modern civil engineering [1]. China’s rapid urbanization over the past few decades has driven a sustained rise in concrete consumption [2]. Meanwhile, the massive demolition of outdated buildings produces enormous construction debris, whose rational disposal and resource recycling have emerged as pressing environmental challenges [3]. Processing waste concrete from construction debris into recycled concrete aggregate (RCA) to replace natural coarse aggregate represents an environmentally friendly and economical approach to resource utilization [4]. By mixing RCA with an appropriate amount of cement and supplementary cementitious materials, recycled aggregate concrete (RAC) with a certain strength and durability can be produced [5]. The recycling of RCA helps reduce reliance on natural coarse aggregates and lowers the carbon emissions of the construction industry, thereby offering significant environmental, economic and social benefits [6].

Despite its promising application prospects, RAC generally exhibits lower mechanical properties than ordinary concrete, which limits its widespread use [7]. To utilize RCA more effectively, researchers have explored various methods, such as adjusting the water-to-cement ratio, employing different mixing procedures [8], or modifying the content, particle size and shape of the aggregates [4,9]; however, the overall improvement has been limited. In addition, various fibers—including steel fibers, basalt fibers, recycled fibers and synthetic fibers—have been introduced to enhance the mechanical performance of RAC [10,11,12,13,14]. Although some fibers do not markedly increase strength, they can effectively improve the toughness and impact resistance of RAC [15]. The incorporation of fibers helps inhibit cracking in concrete caused by loading or shrinkage [16,17]. Numerous studies have found that carbon fiber (CF), as a toughening material, can not only improve the compressive strength, elastic modulus and corrosion resistance of RAC, but also significantly modify its failure mode under compression [18]. However, for RAC containing aged mortar and a complex interfacial transition zone (ITZ), the strengthening mechanism of CF on mechanical properties during compression is particularly complicated, and systematic research at multiple scales is therefore necessary. Meanwhile, researchers have also employed microscopic characterization techniques such as scanning electron microscopy (SEM) [19], X-ray diffraction [20,21] and acoustic emission (AE) [22] to reveal the microstructural characteristics of RAC.

The dynamic behavior of concrete is crucial for structures subjected to dynamic loads, especially in earthquake-prone regions such as China and Japan [23]. Studies have shown that the macroscopic mechanical response of concrete structures under dynamic loading differs significantly from that under static loading, generally exhibiting higher elastic modulus and strength [24,25,26,27]. Consequently, extensive research has been conducted on the dynamic mechanical properties of concrete. He et al. [28] reported that when the strain rate ranges from 10^−5^/s to 10^−2^/s, the biaxial compressive strength of concrete increases linearly with strain rate. Si et al. [29] further found that an increase in strain rate accelerates the evolution of interfacial shear cracks in asphalt concrete, providing a basis for establishing rate-dependent damage models. Wang et al. [30] observed through uniaxial tensile tests that the tensile strength of concrete increases significantly when strain rate is raised from 10^−6^/s to 10^−4^/s. Xiao et al. [31] and Li et al. [32] investigated the dynamic mechanical behavior of RAC with different RCA replacement ratios, and their results indicated that the elastic modulus and peak stress of RAC are markedly enhanced with increasing strain rate; however, the correlation between strain rate and peak strain remains unclear. Although the dynamic mechanical properties of conventional concrete have been extensively studied, the dynamic characteristics of RAC still represent an important research direction.

When carbon fiber (CF) is incorporated into RAC as a reinforcing material, its high tensile strength and aspect ratio enable it to effectively inhibit the initiation and propagation of microcracks through a bridging effect during loading, thereby improving the mechanical performance of RAC under dynamic loads. Huang et al. [33] used fly plate impact tests to systematically evaluate the influence of 0.2% volume fraction of polyacrylonitrile-based short-chopped CF on the dynamic mechanical behavior of mortar within the strain rate range of 10^−3^/s to 10^−2^/s, confirming that the bridging mechanism of CF can effectively delay rapid crack propagation. However, recycled concrete itself is a multi-phase, multi-scale heterogeneous material, and its dynamic mechanical properties depend on the inherent complexity of the material, the variation in CF content, and the difference in strain rate. Therefore, when investigating the mechanical characteristics of carbon fiber-reinforced recycled aggregate concrete (CFRRAC), it is necessary to consider these multiple factors comprehensively to more accurately reveal its dynamic failure mechanisms and strengthening laws.

At present, studies focusing on the individual effects of CF content or strain rate on the mechanical behavior of RAC are relatively abundant. Nevertheless, most existing work has concentrated on macroscopic strength indices and qualitative microscopic analyses, while quantitative investigations of mesoscopic damage mechanisms under different strain rates remain rather limited. Hence, it is essential to explore in depth the evolutionary laws of the mechanical behavior of CFRRAC under the synergistic action of the above factors. As a typical quasi-brittle material, the deformation and failure of concrete are essentially a continuous damage evolution process in which internal initial defects—such as micropores and microcracks—continuously nucleate, initiate, propagate and eventually coalesce. The failure behavior involves cross-scale interactions among disordered and heterogeneous structures at the macroscopic, mesoscopic and microscopic levels [34]. The macroscopic nonlinear stress–strain response of the material is governed by microstructural heterogeneity and nonlinear damage evolution at the microscale. Therefore, investigating the mechanical properties and mesoscopic damage mechanisms of CFRRAC under coupled CF content and strain rate conditions is of considerable research value for revealing the intrinsic physical mechanisms underlying its damage and failure.

Statistical damage mechanics provides an effective approach for elucidating the mesoscopic damage mechanisms of quasi-brittle materials such as concrete. In 1982, Krajcinovic et al. [35] proposed the parallel bar model (PBS) for the uniaxial tensile process of concrete; since then, statistical damage theory has been widely applied in the damage mechanics of concrete, rock and other materials. Bai et al. [36,37,38] established a mesoscopic statistical damage model in which the representative volume element is abstracted as a system of numerous mesoscopic springs or micro-bars obeying specific probability distributions (e.g., Weibull distribution or normal distribution). By deriving statistical evolution equations for the failure of micro-elements, they established a link between mesoscopic damage mechanisms and macroscopic nonlinear stress–strain behavior. The microstructural characteristics of specimens with different CF contents and the strain rate effects further influence the initiation morphology and propagation paths of microcracks during uniaxial compression, thereby affecting the mesoscopic fracture and yielding damage evolution processes. With the aid of mesoscopic statistical damage theory, the roles of the aforementioned factors can be further elucidated.

Although previous studies have extensively investigated the individual effects of CF content [18] or strain rate [31,32] on the mechanical behavior of RAC, and microscopic techniques such as SEM and AE have been employed for qualitative microstructural characterization [19,22], most existing work has focused on macroscopic strength indices and phenomenological observations. Quantitative investigations of mesoscopic damage evolution under the coupled influence of CF content and strain rate remain limited, and the synergistic effects of these two factors on damage characteristic parameters have not been systematically addressed. In this study, uniaxial compression tests were conducted on CFRRAC with different CF contents (0%, 0.15% and 0.3%) and strain rates (10^−5^/s, 10^−4^/s, 10^−3^/s, 10^−2^/s) to obtain complete stress–strain curves, aiming to investigate its dynamic mechanical properties and deformation failure characteristics. Acoustic emission (AE) technology was employed to monitor microcrack propagation in the specimens during uniaxial compression, and scanning electron microscopy (SEM) was used to analyze microstructural morphology. Based on a statistical damage constitutive model, the evolution laws of the mesoscopic damage parameters (*ε*_a_, *ε*_b_, *ε*_h_ and *H*) of CFRRAC under different strain rates are elucidated, thereby establishing an effective correlation between mesoscopic damage mechanisms and macroscopic nonlinear constitutive behavior. This study attempts to provide, the present study provides (1) a quantitative assessment of the synergistic enhancement of CF content and strain rate on the mechanical parameters of CFRRAC; (2) the identification of regular trends in mesoscopic damage characteristic parameters (εa, εb, εh, and H) with varying CF content and strain rate; and (3) the revelation of underlying damage evolution mechanisms via damage spectra, offering a deeper understanding of the rate-dependent failure process of CFRRAC.

## 2. Materials and Methods

### 2.1. Raw Materials

**Figure 1:**
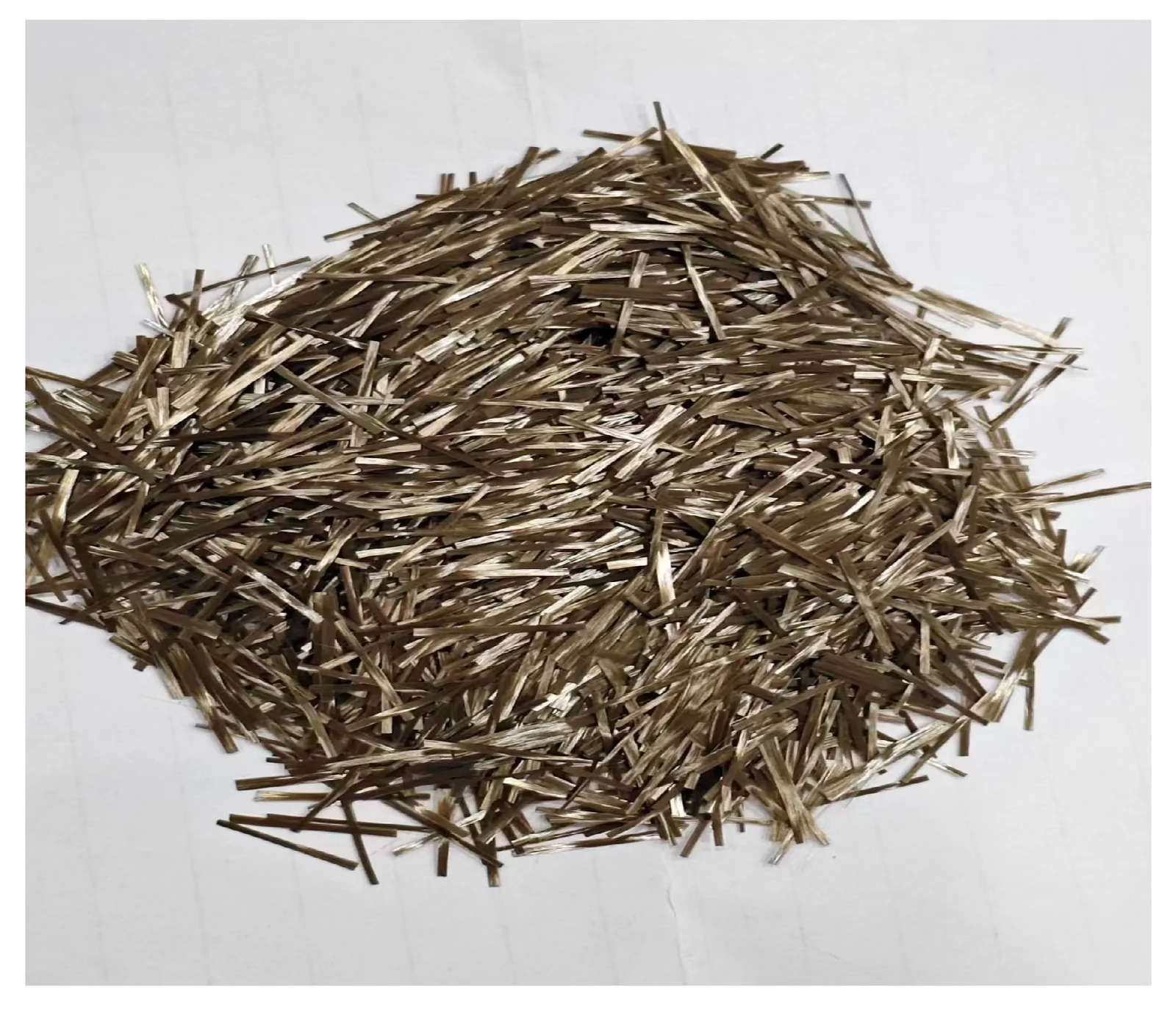
Short-cut CFs.

**Figure 2:**
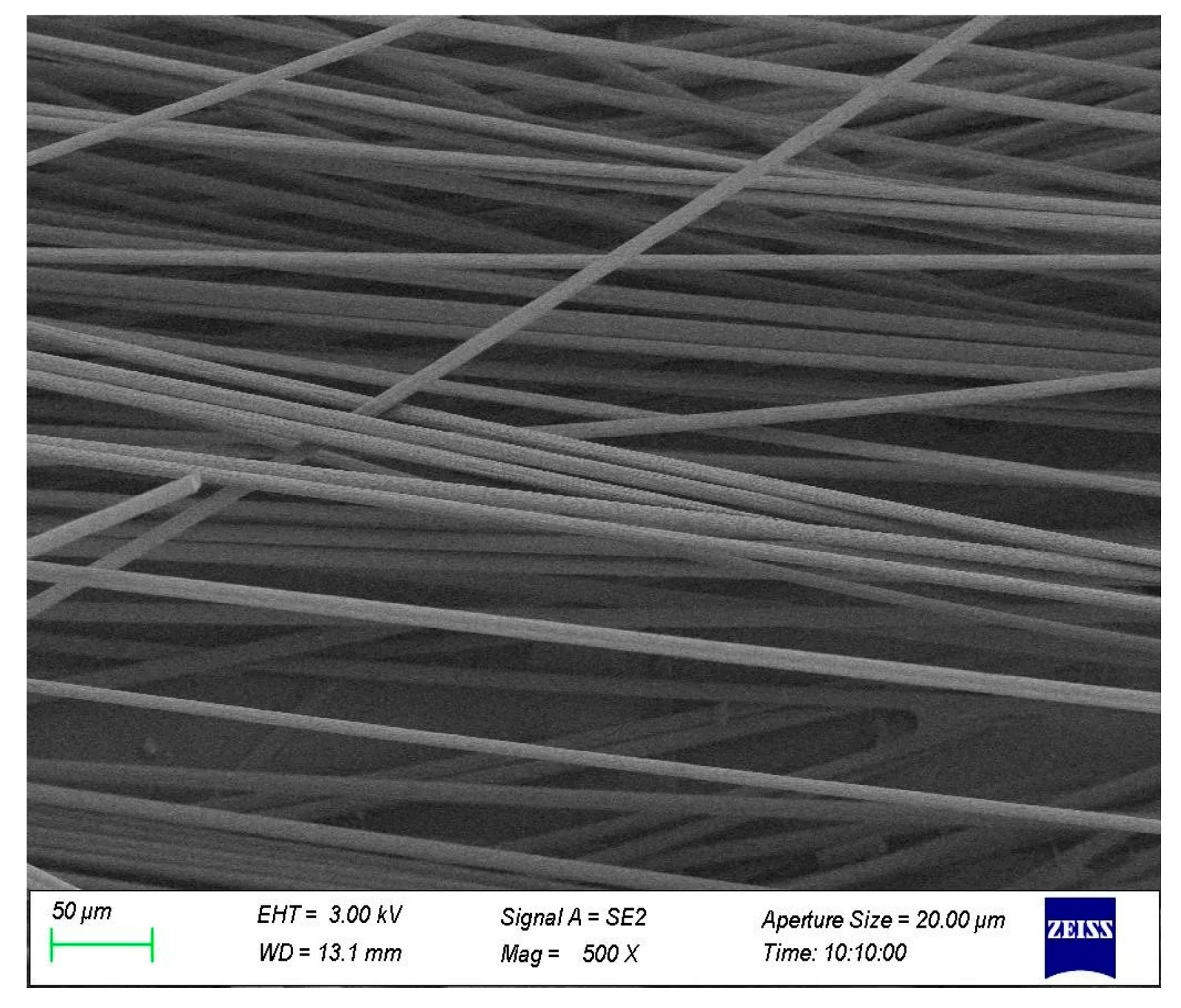
SEM image of CFs.

**Figure 3:**
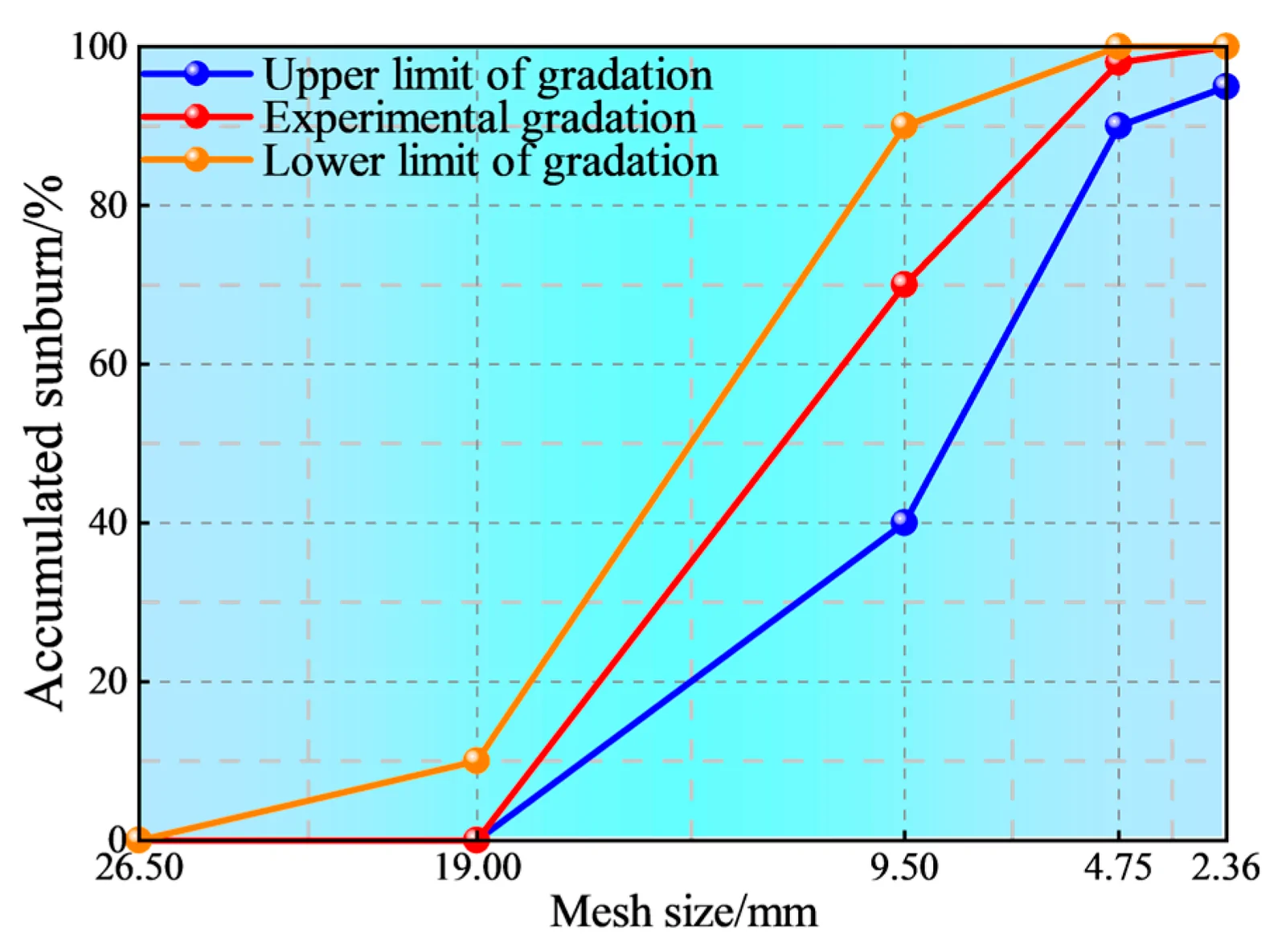
Grain series of RCA.

In this study, the full chemical composition of the cement is presented in Table 1. T700-type short-chopped CF was selected for the experiments, as shown in Figure 1. The performance characteristics of the CF are summarized in Table 2, and a SEM image taken at a magnification of 500 times is displayed in Figure 2. According to the Code for Hydraulic Concrete Tests [39], the fine aggregate consisted of natural river sand with a fineness modulus of 2.75. The RCA used in the experiments was sourced from construction waste in Zhengzhou, China, and its performance indices are listed in Table 3. Figure 3 presents the particle size distribution curve of the RCA, which meets the requirements of the relevant specification.

It should be noted that the RCA was obtained from a local construction waste recycling plant, where the waste concrete originated from mixed demolition sites. As is common in recycled aggregate studies using commercially available materials, the strength grade of the parent concrete could not be reliably identified. Nevertheless, the crushing value (12.39%) and water absorption (5.89%) provided in Table 3 indirectly reflect the mechanical quality of the RCA.

### 2.2. Mix Proportion Design and Specimen Preparation

In the experiments, the volume fractions (*V*_f_) of CF were set to 0%, 0.15% and 0.3%. The CF was added to RAC specimens with dimensions of 100 mm × 100 mm × 100 mm by first dry-mixing the fibers with the aggregates and cement, followed by the addition of water and other admixtures (i.e., the cement content remained unchanged as shown in Table 4). It should be noted that Zhang [38] found that *V*_f_ = 0.3% is considered the optimal CF content, which is consistent with the recommended content given by Huang et al. [29]. Considering that the diameter of a single CF filament is very small (7 μm) and that the fiber is somewhat hydrophobic in nature, hydroxypropyl methyl cellulose was used at a dosage of 0.4% to reduce agglomeration and aggregation of the CF, thereby achieving uniform dispersion. In addition, tributyl phosphate was added at a concentration of 0.1% (by weight of cement) to eliminate air bubbles generated during the fiber dispersion process. The aged cement mortar covering the outer surface of the RCA has a greater water absorption capacity than that of NCA. Therefore, during the mix proportion design, extra water derived from the RCA was added according to a water-to-cement ratio of 0.46 [40]. Meanwhile, a polycarboxylate superplasticizer with a water-reducing rate of 18% was incorporated into the mixture at a dosage of 0.3% (by weight of cement) to improve the workability of the fresh concrete. The mixed proportions of the specimens with different CF contents are presented in Table 4.

**Figure 4:**
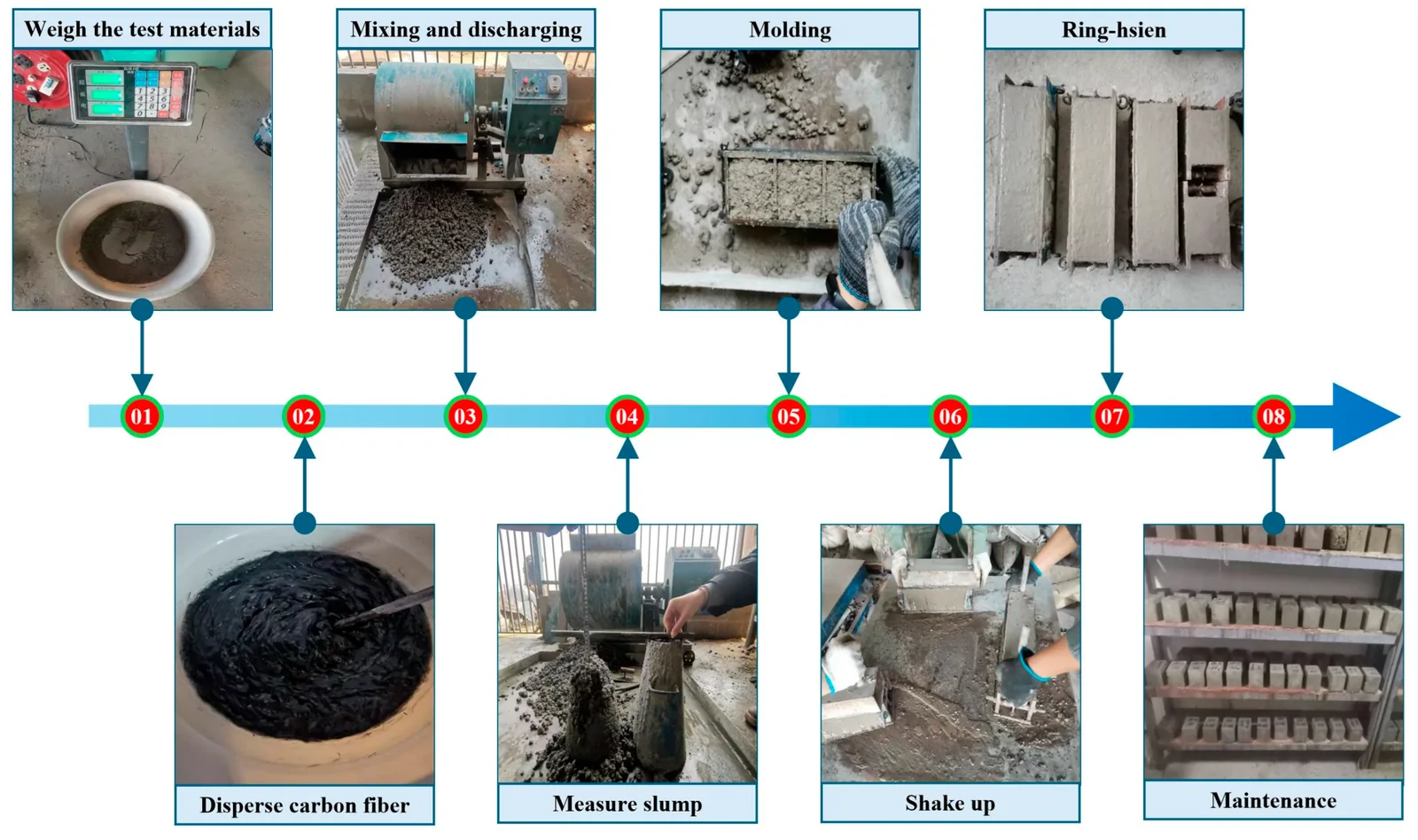
Flow chart of specimen preparation.

Specimen casting was carried out in accordance with the Standard for Test Methods of Performance of Ordinary Concrete Mixtures (GB/T 50080-2016) [41], and the specific procedure is illustrated in Figure 4. The test materials were weighed according to the mix proportion and dry-mixed in a mixer for 60 s while the CF dispersion was being prepared. Subsequently, the mixer was started, and the CF dispersion was slowly added during the 180 s mixing process to obtain the carbon fiber-reinforced concrete mixture. After verifying that the slump met the requirement, the molding process was initiated: the mixture was placed into the molds in three layers, and each layer was vibrated on a vibrating table for 20 s and compacted with a tamping rod. The surface was then levelled with a scraper and covered with plastic film. After curing in the laboratory for 1 day, the specimens were demolded, marked, and transferred to a standard curing room. Following 28 days of curing, they were removed and kept for subsequent use.

### 2.3. Test Methods

#### 2.3.1. Uniaxial Compression

**Figure 5:**
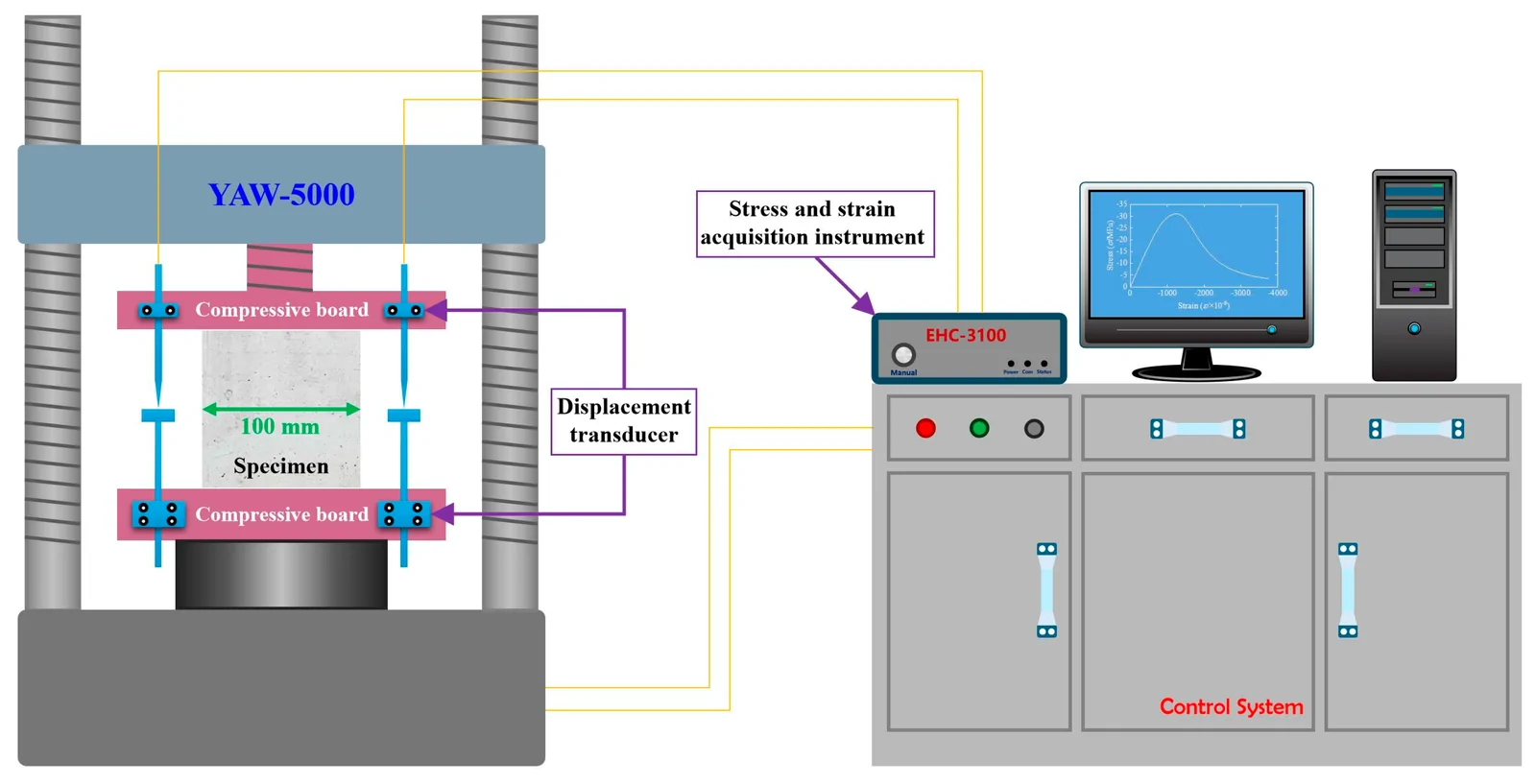
Schematic diagram of uniaxial compression test.

To systematically obtain the fundamental mechanical parameters of recycled aggregate concrete under compressive loading, standardized testing protocols have been extensively adopted in previous studies [42]. In this study, a YAW-5000 microcomputer-controlled electro-hydraulic servo press (Sansimex Technology Co., Ltd., Shanghai, China) was employed as the uniaxial compression testing device. Prior to loading, the contact surfaces of the specimens were waxed to ensure smoothness, thereby reducing stress concentration-induced non-uniform stress distribution and friction between the platens and the specimen. The loading process consisted of two stages, preloading and formal loading, to avoid the influence of internal defects (such as loose end fractures) on testing accuracy. Initially, preloading was performed using a displacement-controlled method at an average speed of 5 mm/min, and formal loading commenced once the load reached 5 kN. The four loading rates of the press—0.06 mm/min, 0.6 mm/min, 6 mm/min and 60 mm/min—corresponded to four strain rates of 10^−5^/s, 10^−4^/s, 10^−3^/s and 10^−2^/s, respectively. The quasi-static loading rate was set to 0.06 mm/min [37]. The testing machine possessed high-precision loading control capabilities. A high-precision displacement transducer was used to measure the longitudinal deformation of the specimens, to obtain accurate stress–strain curves for each specimen, as shown in Figure 5.

#### 2.3.2. Scanning Electron Microscope

To investigate the microstructure of CFRRAC, concrete specimens with two CF volume fractions (0% and 0.3%) were selected for scanning electron microscopy (SEM) examination. The specific procedures were as follows: fragments with regular shapes, smooth surfaces and dimensions of approximately 1 cm were chosen as samples. The samples were dried at 60 °C for 24 h and then placed into clearly labelled sample bags. Subsequently, the samples were vacuum-treated and sputter-coated with gold to enhance the observation clarity. The morphological features of the characteristic micro-areas were captured using SEM, and images at various magnifications were acquired as required. The above procedures yielded detailed microstructural images, providing a basis for analyzing the internal components and microstructure of CFRRAC.

#### 2.3.3. Acoustic Emission

Acoustic emission (AE), as a non-destructive testing method, has been widely used for damage monitoring of concrete structures. When materials undergo deformation or damage under loading, they release energy in the form of elastic waves, and this phenomenon is referred to as AE. The deformation and failure process of concrete is closely related to AE activity: under external loading, concrete successively undergoes deformation, expansion, cracking, coalescence and ultimately instability failure. AE monitoring was performed using a SAEU3H acoustic emission system (Beijing Soundwel Technology Co., Ltd., Beijing, China) with a sampling rate of 1 MHz. R6α resonant sensors with a frequency range of 50–200 kHz were employed. The sensors were coupled to the specimen surface using Vaseline as a couplant and secured with rubber bands. A preamplification of 40 dB was applied, and the threshold was set at 40 dB to filter background noise. This threshold was selected based on preliminary tests and is consistent with values commonly used in acoustic emission studies of concrete. Two sensors were symmetrically placed on opposite side surfaces of each specimen to ensure reliable signal detection. Prior to each test, the sensors were calibrated using a pencil lead break test to verify proper coupling.

Macroscopically, this is manifested as a reduction in load-bearing capacity and final failure, while mesoscopically, it is accompanied by energy release during the initiation and propagation of microcracks. The generated elastic waves propagate to the specimen surface and induce mechanical vibrations. After acquisition and processing of these signals, AE characteristic parameters can be obtained, based on which the damage evolution and crack propagation process of concrete can be analyzed. In this study, AE testing was conducted simultaneously with the uniaxial compression tests. During the test, the press was operated at a quasi-static loading rate, and the hit parameters and waveform sampling were set according to the AE instrument instructions. After applying Vaseline to the AE sensors, they were fixed to the side surfaces of the specimens using rubber bands. Throughout the loading process, the press and AE monitoring were kept synchronized. When the load dropped to approximately 30% of the peak load, both loading and data acquisition were stopped simultaneously, and the test was terminated.

## 3. Results and Analysis

### 3.1. Analysis of Mechanical Parameters

**Figure 6:**
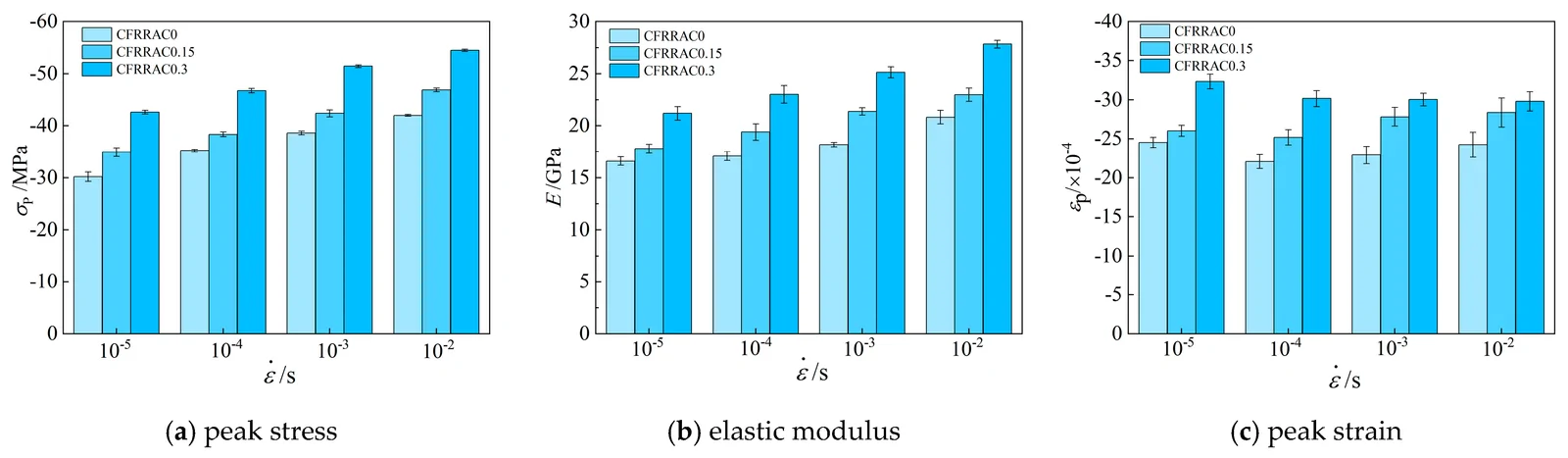
Mechanical characteristic parameters of CFRRAC.

From the uniaxial compression tests, the peak stress, elastic modulus, and peak strain of CFRRAC with three different CF contents (0%, 0.15%, and 0.3%) at four strain rates ($\dot{\varepsilon }$: 10^−5^/s, 10^−4^/s, 10^−3^/s, and 10^−2^/s) were obtained, as presented in Figure 6. Each data point represents the average value of three replicate specimens.

(1) Peak stress

Figure 6a indicates that the peak stress of the specimens is positively correlated with CF content. At $\dot{\varepsilon }$ = 10^−5^/s, the peak stresses of the CFRRAC0, CFRRAC0.15 and CFRRAC0.3 specimens were −30.84 MPa, −35.23 MPa and −42.45 MPa, respectively, corresponding to increases of 14.2% and 37.7%. The improvements are substantially larger than the experimental variability, confirming the effectiveness of CF addition. Peak stress increases consistently with CF content across all strain rates, with the most pronounced enhancement observed at the highest CF content of 0.3%. This improvement is primarily attributed to the bridging effect of CF, which transfers and redistributes internal tensile stresses, thereby enhancing the energy absorption capacity and delaying macroscopic failure. The strain rate sensitivity of peak stress is also evident: taking CFRRAC0.3 as an example, peak stress rises steadily as strain rate increases from 10^−4^/s to 10^−2^/s, reflecting the combined influence of inertial effects and the Stefan effect, which restrict microcrack propagation under faster loading. Moreover, the magnitude of strain rate sensitivity tends to increase with CF content, suggesting that the fiber bridging effect and the strain rate effect act synergistically to improve the load-bearing capacity of CFRRAC. It is evident that the appropriate incorporation of carbon fibers can significantly enhance the compressive strength of RAC.

For the same CF content, peak stress increases steadily with rising strain rate. Taking the CFRRAC0.3 specimens as an example, peak stress rises from 42.45 MPa at 10^−5^/s to 55.71 MPa at 10^−2^/s, an enhancement of approximately 31%, confirming the significant strain rate sensitivity of CFRRAC. This behavior is governed by the inertial effect and the Stefan effect: under faster loading, microcrack propagation is constrained by time limitations, forcing cracks to follow paths with higher energy dissipation, while the Stefan effect introduces viscous resistance in thin water films within narrow pores, effectively delaying crack surface separation. Comparing the three groups, the strain rate sensitivity of peak stress tends to be more pronounced at higher CF contents, with CFRRAC0.3 exhibiting the greatest enhancement. This suggests that fiber bridging and strain rate effects act synergistically to improve the load-bearing capacity of CFRRAC.

(2) Elastic modulus

As shown in Figure 6b, the elastic modulus increases with both CF content and strain rate. At the quasi-static strain rate of 10^−5^/s, the value rises from 16.60 GPa for plain RAC to 21.20 GPa for CFRRAC with 0.3% CF, demonstrating a clear stiffening effect. This enhancement is attributable to the strong interfacial bonding between fibers and the RAC matrix, which restricts microcrack initiation at early loading stages [43].

For the same CF content, the elastic modulus also increases monotonically with rising strain rate. Taking the CFRRAC0.3 specimens as an example, the elastic modulus reaches 27.83 GPa at 10^−2^/s, representing an enhancement of approximately 31% over the quasi-static value. The CFRRAC0.15 and CFRRAC0 specimens show similar but less pronounced increases. These results indicate that the strain rate stiffening effect is consistently present, and the incorporation of CF further enhances this strain rate sensitivity.

(3) Peak strain

As illustrated in Figure 6c, peak strain is generally enhanced by the addition of CF. At the quasi-static strain rate of 10^−5^/s, the value increases from −2.451 × 10^−3^ for plain RAC to −3.232 × 10^−3^ for CFRRAC with 0.3% CF, indicating improved deformation capacity before failure. This is consistent with the ductilizing effect of fiber bridging, which allows for greater inelastic deformation prior to peak load.

For the same CF content, however, peak strain shows no consistent dependence on strain rate: for CFRRAC0 and CFRRAC0.15, it first decreases and then increases with rising strain rate, whereas for CFRRAC0.3, it decreases monotonically. This suggests that the strain rate sensitivity of peak strain is modulated by CF content, and the underlying mechanism may involve competing effects between accelerated microcrack propagation and the Stefan effect, superimposed on the inherent material discreteness [44]. Similar observations have been reported in previous studies [45].

### 3.2. Failure Mode

**Figure 7:**
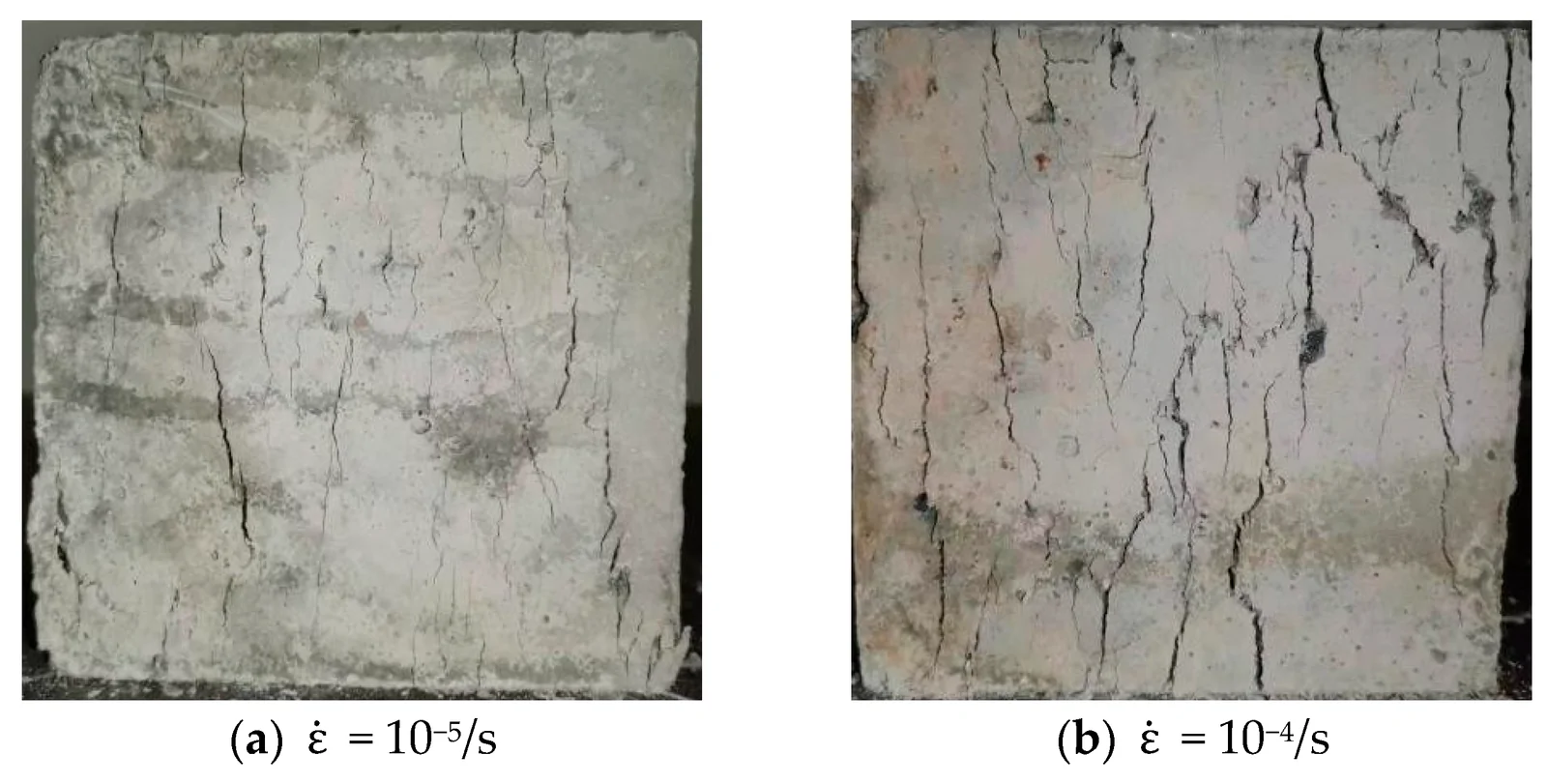

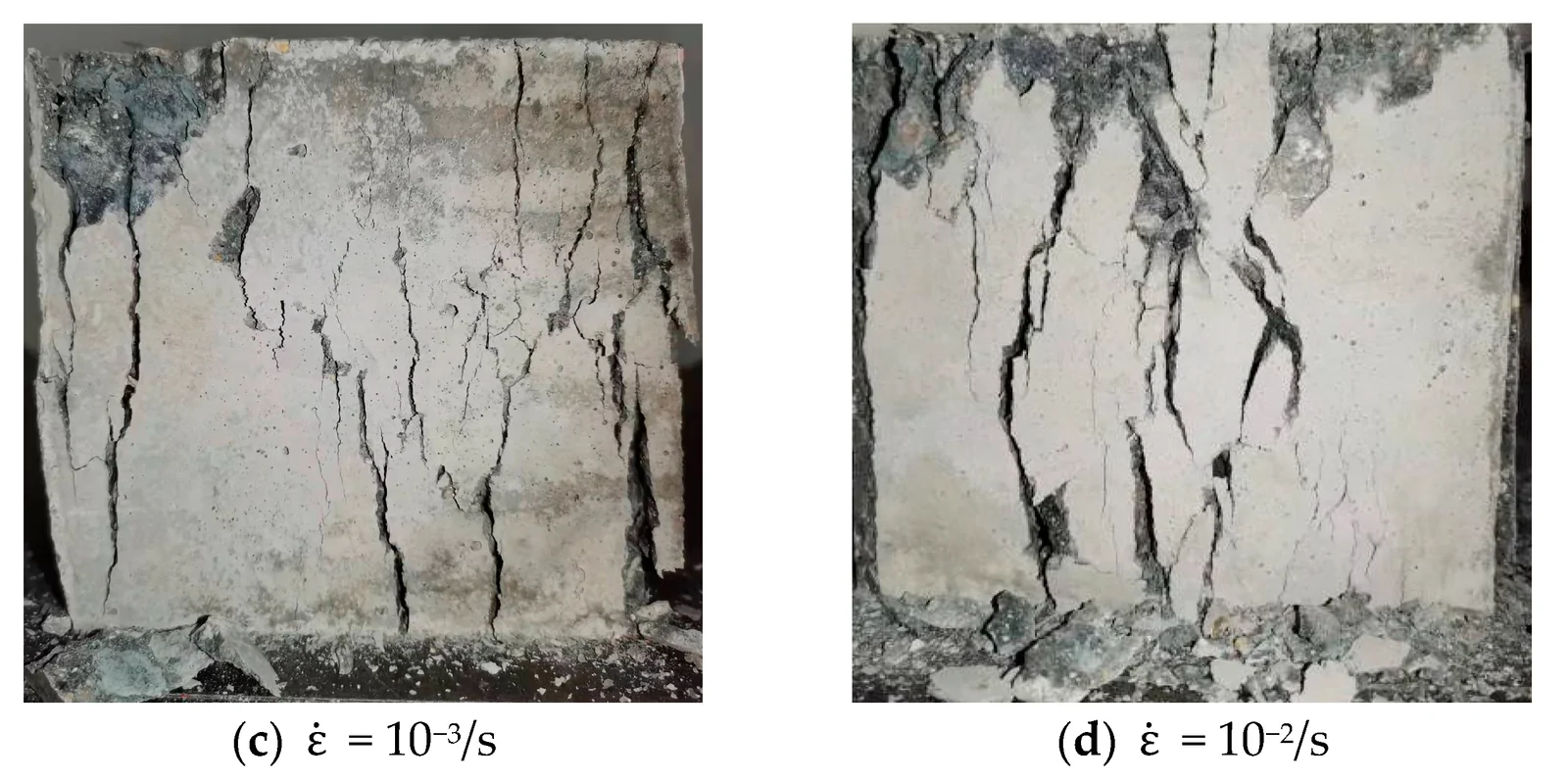
Failure characteristic diagram of CFRRAC0.3.

Figure 7 presents the compressive failure morphologies of the CFRRAC0.3 specimens at different $\dot{\varepsilon }$. The failure characteristics of the specimens with all CF content exhibited similar patterns. With increasing $\dot{\varepsilon }$, the directions of applied loading and crack propagation became nearly parallel. The specimens did not exhibit typical brittle failure features. This is because the uniformly distributed CF exerted a reinforcing effect on the concrete matrix, which inhibited microcrack propagation to a certain extent and made it more likely for the specimens to reach a uniform stress state. The width and number of surface cracks varied with $\dot{\varepsilon }$. At $\dot{\varepsilon }$ = 10^−5^/s and $\dot{\varepsilon }$ = 10^−4^/s, the failure characteristics of the specimens were similar, with cracks uniformly distributed on the surface, relatively small in width and large in number. Few mortar fragments spalled off the specimen surface. In contrast, at $\dot{\varepsilon }$ = 10^−3^/s and $\dot{\varepsilon }$ = 10^−2^/s, the surface cracks became noticeably wider and fewer in number, with more penetrating cracks running from the top to the bottom of the specimens. These penetrating cracks were longer and straighter, and more mortar fragments spalled from the surface. In addition, slight cracking sounds were heard during compression, which originated from the fracture of RCA within the specimens as well as the pull-out and fracture of CF. The differences in failure characteristics under different $\dot{\varepsilon }$ are mainly related to the loading rate and the propagation time of microcracks. At lower $\dot{\varepsilon }$, the loading was slow and lasted for a longer duration, allowing sufficient time for microcracks to propagate along the weak ITZ within the internal structure. At higher $\dot{\varepsilon }$, however, the loading duration was short, and microcracks did not have enough time to fully propagate; they thus tended to cut through some of the aggregates, leading to the pull-out or fracture of CF.

### 3.3. SEM Results Analysis

**Figure 8:**
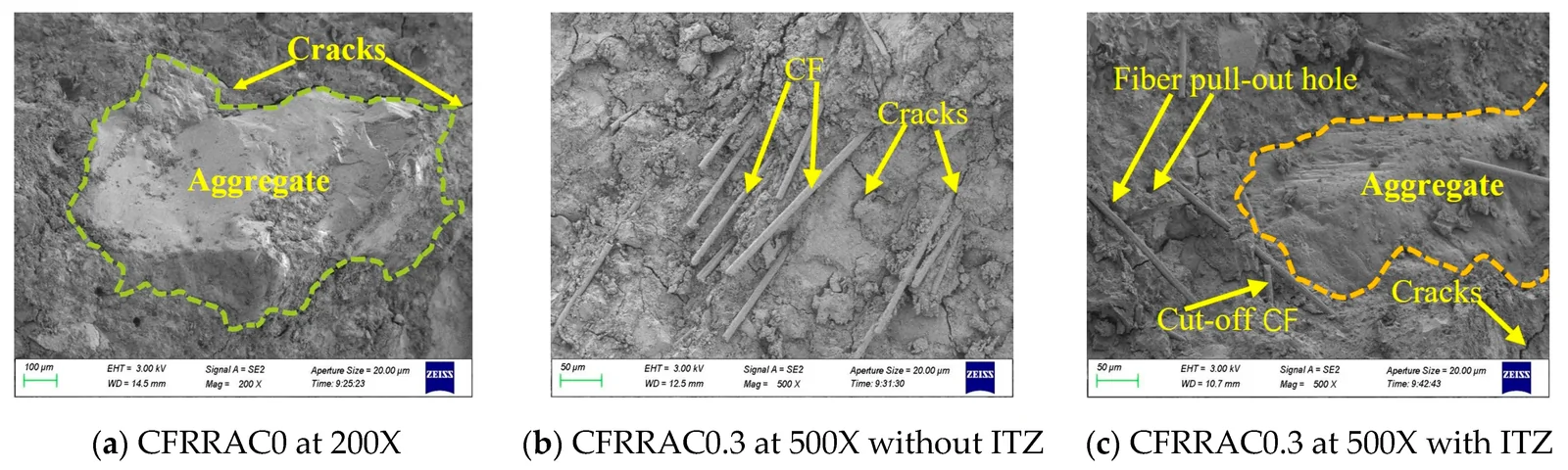
Microstructure of CFRRAC0% and CFRRAC0.3% samples.

To investigate the mechanical strengthening mechanism of CFRRAC, small fragments were collected from the CFRRAC0 and CFRRAC0.3 specimens after quasi-static strain rate compression tests for SEM examination, with the CFRRAC0 specimens serving as the control group to analyze the improvement effect of CF on the performance of CFRRAC0.3. Figure 8 presents the SEM images of the two types of specimens. The ITZ between RCA and cement paste is regarded as a weak region in concrete, owing to the difficulty for cement paste to effectively adhere to the RCA surface [46]. Figure 8a shows the propagation of microcracks within the ITZ: during uniaxial compression, microcracks gradually propagated, extended and interconnected along low-resistance paths, eventually forming penetrating cracks that led to the loss of load-bearing capacity and final failure of the concrete. After the incorporation of CF, as shown in Figure 8b, the CF was uniformly distributed in the cement paste and could effectively inhibit the initiation and propagation of microcracks, delaying their evolution into macroscopic cracks, thereby enhancing the mechanical properties. The failure modes of CFRRAC mainly included fiber pull-out and fiber fracture. Owing to its relatively high elastic modulus and tensile strength, CF exerted a bridging effect within the matrix, undertaking and transferring the stresses induced by crack propagation, effectively reducing the generation of cracks and consequently reinforcing the mechanical performance of concrete [47].

### 3.4. AE Results Analysis

**Figure 9:**
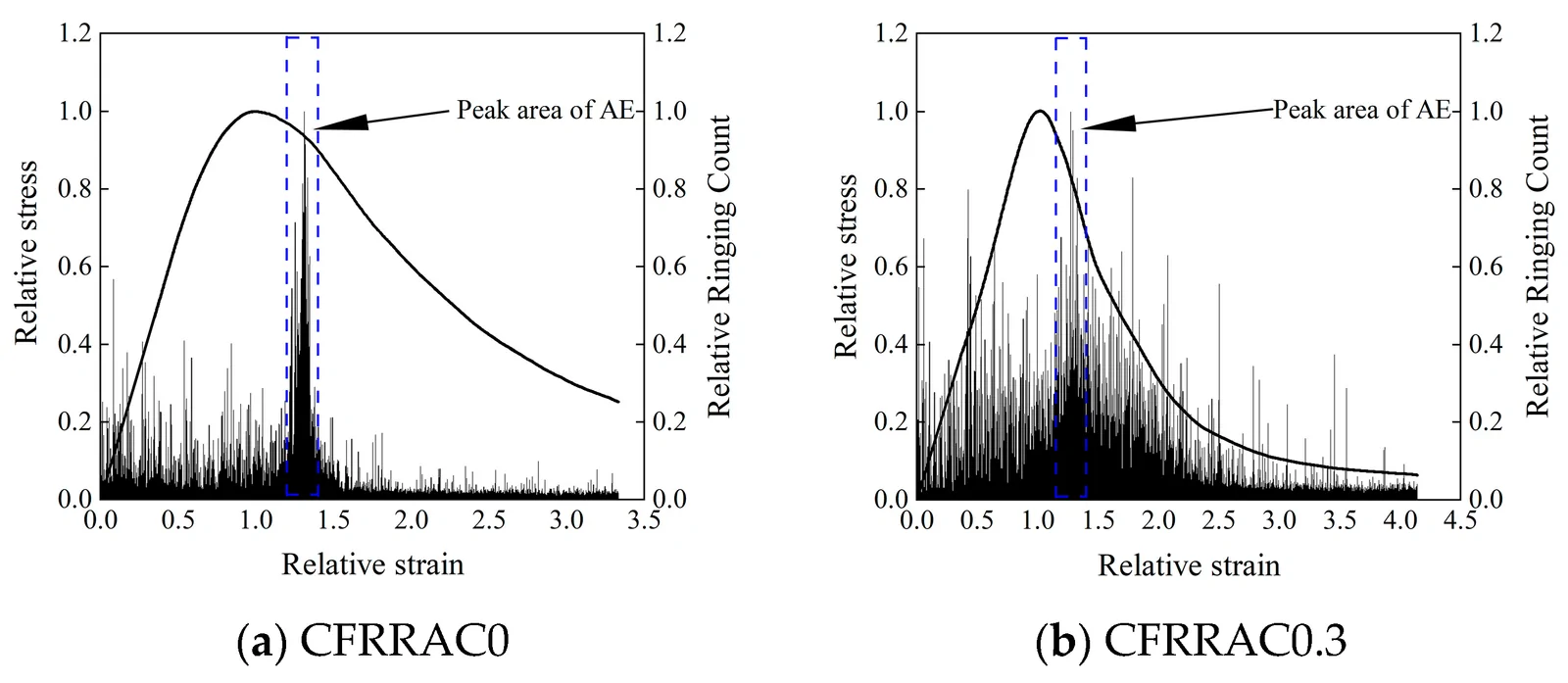
Ringing count and stress–strain curves.

AE signal characteristics are closely related to the formation and propagation of internal cracks during the deformation and failure process of materials and can effectively reflect the damage evolution of materials under different conditions. Among the AE characteristic parameters, the ring-down count is commonly used to analyze and monitor the damage development of specimens. The ring-down count is defined as the number of times that an oscillating signal exceeds a preset threshold and can be used to characterize the intensity of AE signals. Figure 9 presents the normalized relative stress versus relative strain curves and the normalized relative ring-down count versus relative strain curves. It can be observed that the trends of relative stress and relative ring-down count with increasing relative strain are generally consistent. Based on this trend, the entire loading process can be divided into four stages: In the initial compaction stage, after preloading, the internal pores of the specimen are gradually compacted, with only a small number of microcracks generated, and the relative ring-down count remains relatively low. Upon entering the elastic compression stage, as the stress gradually increases, internal cracks propagate stably, and the relative ring-down count rises correspondingly. When the curve passes the peak stress and enters the strain-softening stage, the relative ring-down count continues to increase and reaches its maximum value. At this point, microcracks progressively coalesce into macroscopic cracks, and the specimen reaches the critical state. The mesoscopic load-bearing skeleton has been adjusted to its optimum through stress redistribution and can no longer sustain further loading, after which the specimen enters the local failure stage. Following the critical state, the load-bearing capacity of the specimen continuously decreases, the relative stress gradually diminishes, and the relative ring-down count also falls back to a relatively low level. In this study, crack initiation was identified as the point where the relative ring-down count showed a sustained increase above the background level, which consistently corresponded to the end of the compaction stage (i.e., the onset of the elastic compression stage). This criterion was adopted because the ring-down count effectively reflects the intensity of microcracking events. However, it should be noted that more detailed analyses using other AE parameters (e.g., cumulative hits, energy, RA/AF values) were not performed, and this will be addressed in future work. In addition, it can be observed from Figure 9 that the AE peak region does not correspond to the peak stress point on the stress–strain curve but rather lags the peak stress. This position coincides exactly with the critical state in the statistical model. When the specimen reaches the critical state, microcrack propagation becomes more fully developed and evolves into macroscopic cracks, at which point the AE activity is most intense and the signal strength is highest.

It should be noted that the AE ring-down count primarily characterizes the instantaneous intensity of microcracking events rather than the cumulative energy dissipation over the entire loading history. The total area under the stress–strain curve is governed by the combined effects of fracture damage and yielding damage, as well as the CF bridging toughening effect, which complicates a direct quantitative correlation between AE activity and the total absorbed energy. Furthermore, as discussed above, the AE peak region lags behind the peak stress and coincides with the critical state, indicating that AE activity is most pronounced during the transition from distributed damage to localized failure.

### 3.5. Stress–Strain Curves

**Figure 10:**
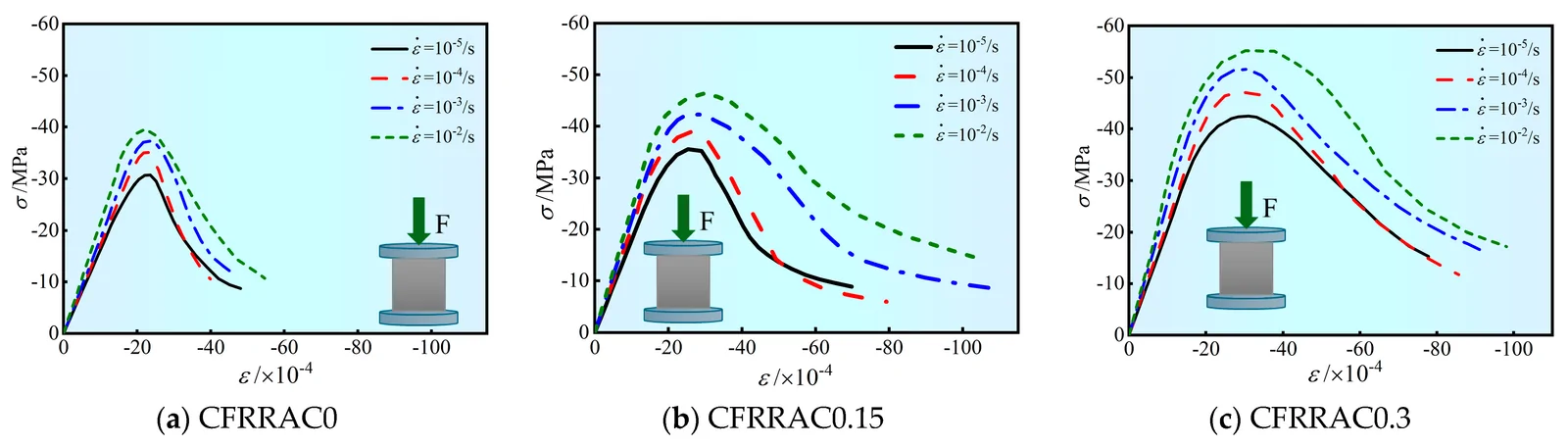
Stress–strain curves of CFRRAC.

Figure 10 presents the stress–strain curves of CFRRAC with three CF contents (0%, 0.15% and 0.3%) at different strain rates. The uniaxial compressive stress–strain curves of all specimens exhibit similar overall shapes, each consisting of an ascending branch and a descending branch, with a smooth and continuous transition between the two stages. An increase in CF content significantly modifies the curve morphology: the slope of the ascending branch increases, and the peak point shifts upward and to the right, reflecting simultaneous improvements in material stiffness, strength and peak strain. The descending branch becomes gentler, indicating that the ductility of the failure process is enhanced. This is attributed to the fact that CF improves the plastic deformation capacity of CFRRAC and ameliorates the brittle failure characteristics of concrete [48]. For the same CF content, an increase in strain rate also induces regular changes in the curve morphology. As the strain rate rises, the ascending branch becomes steeper, and the peak stress and elastic modulus of the specimens increase accordingly. The descending branches of the curves remain roughly parallel to each other.

## 4. Analysis of Mesoscopic Damage Mechanism

### 4.1. Statistical Damage Model

**Figure 11:**
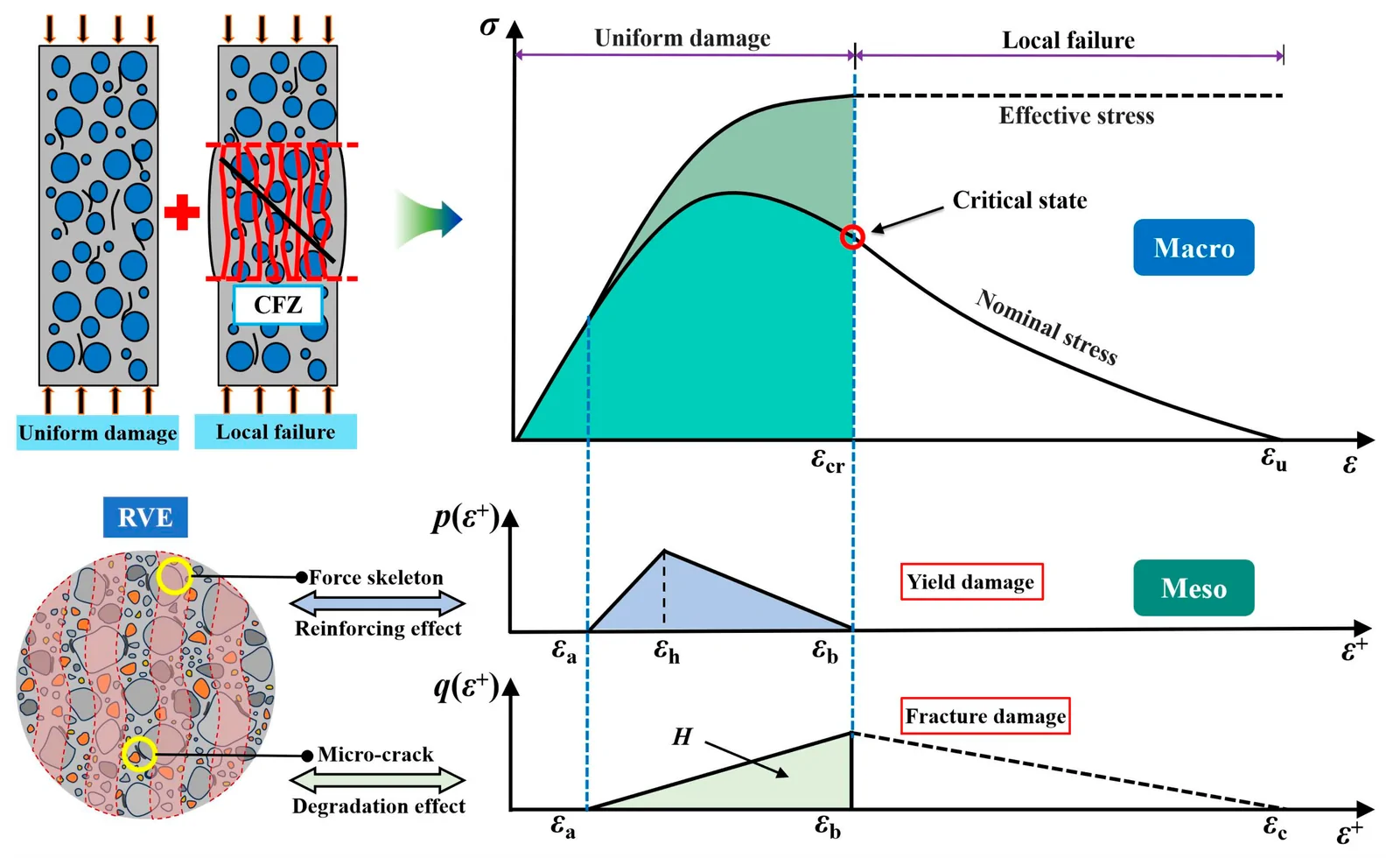
Relationship between macro-constitutive behavior and meso-damage mechanism [50].

Bai et al. and Yuan et al. [49,50,51,52] introduced the concept of “latent mechanical performance,” regarding the deformation and failure of concrete as a self-organizing behavior in which the load-bearing skeleton of the material microstructure undergoes optimal adjustment to accommodate changes in the external loading environment. During the deformation and failure process, two competing effects exist within the microstructure, namely the “deterioration effect” and the “strengthening effect,” which correspond to microcrack initiation and propagation and the optimal adjustment of the load-bearing skeleton, respectively. On this basis, a statistical damage model for concrete under uniaxial compression was established, as shown in Figure 11. The lateral tensile damage induced by the Poisson effect governs the damage evolution in the compression direction. An equivalent tensile damage strain ε+ is introduced, satisfying ε+ = −vε, where v is Poisson’s ratio (taken as 0.2) and ε is the strain in the compressive direction. The compressive process of concrete is divided into two stages: the homogeneous damage stage and the local failure stage, with the junction between them termed the critical state. Figure 11 illustrates the evolution of nominal stress and effective stress. During the homogeneous damage stage, the nominal stress first increases and then decreases, while the effective stress increases monotonically. When the critical state is reached, the effective stress attains its maximum value, and the specimen subsequently enters the local failure stage. The macroscopic nonlinear stress-strain behavior of concrete results from the combined action of two mesoscopic damage modes, namely fracture and yielding. Fracture damage reflects microcrack propagation, whereas yielding damage reflects the optimal adjustment of the microstructural load-bearing skeleton, corresponding to the “deterioration” and “strengthening” effects, respectively. The functions *p*(*ε*^+^) and *q*(*ε*^+^) denote the probability density functions of mesoscopic fracture damage and yielding damage, respectively. *ε*_a_ and *ε*_b_ are characteristic strains corresponding to *ε*+, representing the initial damage strain and the maximum yielding damage strain, respectively, satisfying *ε*_a_ = −*vε*_0_ and *ε*b = −*vε*_cr_, where *ε*_cr_ is the compressive strain at the critical state. The parameters *ε*b and *ε*h are the strains corresponding to the peaks of *p*(*ε*^+^) and *q*(*ε*^+^), respectively. The constitutive relation corresponding to the homogeneous damage stage can be expressed as follows:

In the uniform damage stage, the constitutive relationship is expressed as follows:

*σ* = *E*(1 − *D*_y_)(1 − *D*_R_)*ε* (1)

*σ*_E_ = *E*(1 − *D*_y_)*ε* (2)

(3) $$D_{y}=\int_{0}^{\varepsilon^{+}}p\left(\varepsilon^{+}\right)d\varepsilon^{+}-\frac{\int_{0}^{\varepsilon^{+}}p\left(\varepsilon^{+}\right)\varepsilon^{+}d\varepsilon^{+}}{\varepsilon^{+}}$$

(4) $$D_{R}=\int_{0}^{\varepsilon^{+}}q\left(\varepsilon^{+}\right)d\varepsilon^{+}$$

(5) $$E_{v}=\int_{0}^{\varepsilon^{+}}p\left(\varepsilon^{+}\right)d\varepsilon^{+}$$

(6) $$q(\varepsilon +)=\left\{\begin{array}{cc} 0 & \left(\varepsilon^{+}\leq \varepsilon_{a}\right)\\ \frac{2H\left(\varepsilon^{+}-\varepsilon_{a}\right)}{{\left(\varepsilon_{b}-\varepsilon_{a}\right)}^{2}} & \left(\varepsilon_{a}<\varepsilon^{+}\leq \varepsilon_{b}\right) \end{array}\right.$$

(7) $$p(\varepsilon +)=\left\{\begin{array}{cc} 0 & \left(\varepsilon^{+}\leq \varepsilon_{a}\right)\\ \frac{2\left(\varepsilon^{+}-\varepsilon_{a}\right)}{\left(\varepsilon_{h-}\varepsilon_{a}\right)\left(\varepsilon_{b}-\varepsilon_{a}\right)} & \left(\varepsilon_{a}<\varepsilon^{+}\leq \varepsilon_{h}\right)\\ \frac{2\left(\varepsilon_{b}-\varepsilon^{+}\right)}{\left(\varepsilon_{b}-\varepsilon_{h}\right)\left(\varepsilon_{b}-\varepsilon_{a}\right)} & \left(\varepsilon_{h}<\varepsilon^{+}\leq \varepsilon_{b}\right) \end{array}\right.$$

*H* = *D*_R_(*ε*_b_) (8)

where *σ* and *σ*_E_ represent the nominal stress and the effective stress, respectively; *E* is the initial elastic modulus; *D*_R_ and *D*_y_ are the fracture damage variable and the yielding damage variable, respectively; the parameter *E*_v_ ranges from 0 to 1, which is used to quantify the degree of optimization of the load-bearing skeleton and is associated with mesoscopic yielding damage; and *H* denotes the fracture damage value at the critical state.

The advantages of this model are threefold: (1) it is capable of characterizing the two-stage feature of distributed damage accumulation and local catastrophe; (2) it can reflect the cumulative process of damage from “quantitative change” to “qualitative change” during the homogeneous damage stage; and (3) more importantly, this model can reasonably explain the hysteresis phenomenon of the critical state of local catastrophe as observed in previous studies [53,54,55]. That is, the critical state corresponds to the maximum effective stress state, and its occurrence lags the peak nominal stress state. When the critical state is reached, the microstructural load-bearing skeleton has been adjusted to its optimum, and the latent mechanical performance has been exploited to its limit; subsequently, the specimen enters the local catastrophe stage.

### 4.2. Analysis of Damage Mechanism

The mesoscopic damage parameters, including $R_{E}$, *ε*_a_, *ε*_b_, *ε*_h_ and *H*, were determined from the stress–strain curves of CFRRAC. $R_{E}=\frac{E_{i}}{E_{0}}$ reflects the strain rate effect on elastic modulus, where $E_{0}$ is the modulus at $\dot{\varepsilon }$ = 10^−5^/s and $E_{i}$ at other strain rates. The parameters *ε*_a_, *ε*_b_, *ε*_h_ and *H* were obtained by multivariate regression using the MATLAB (2024a) Genetic Algorithm Toolbox, and the results are listed in Table 5.

**Figure 12:**
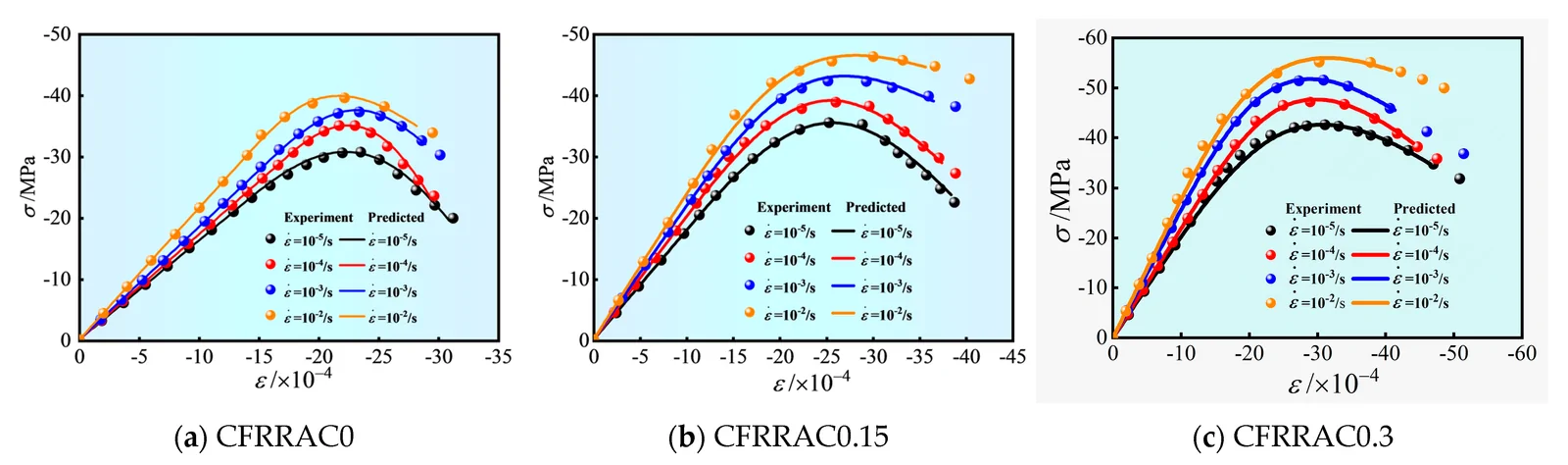
Nominal stress–strain curves.

**Figure 13:**
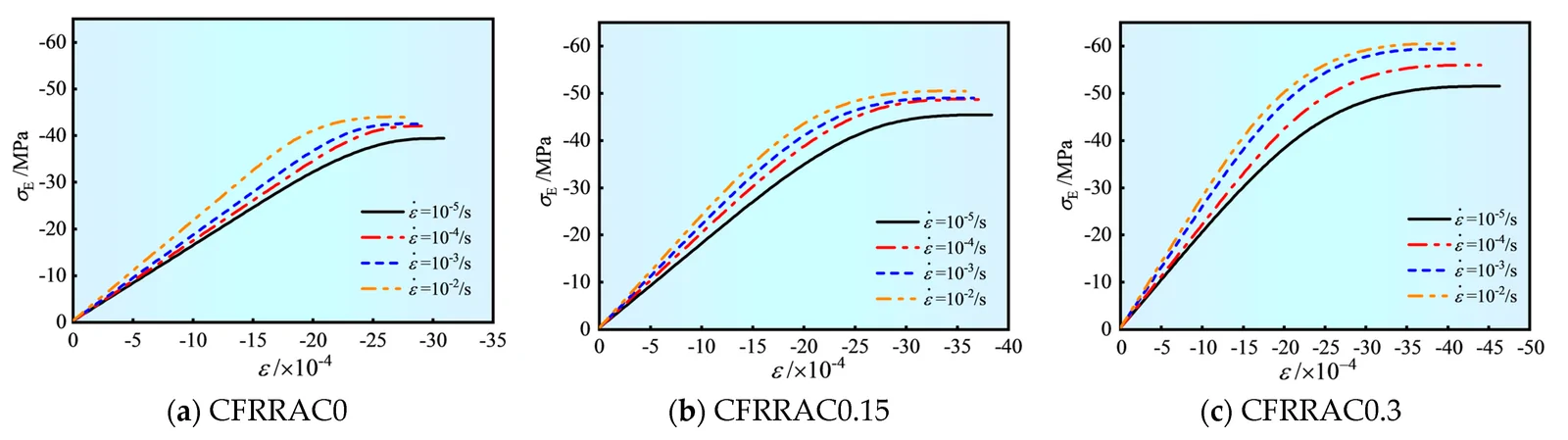
Effective stress–strain curves.

**Figure 14:**
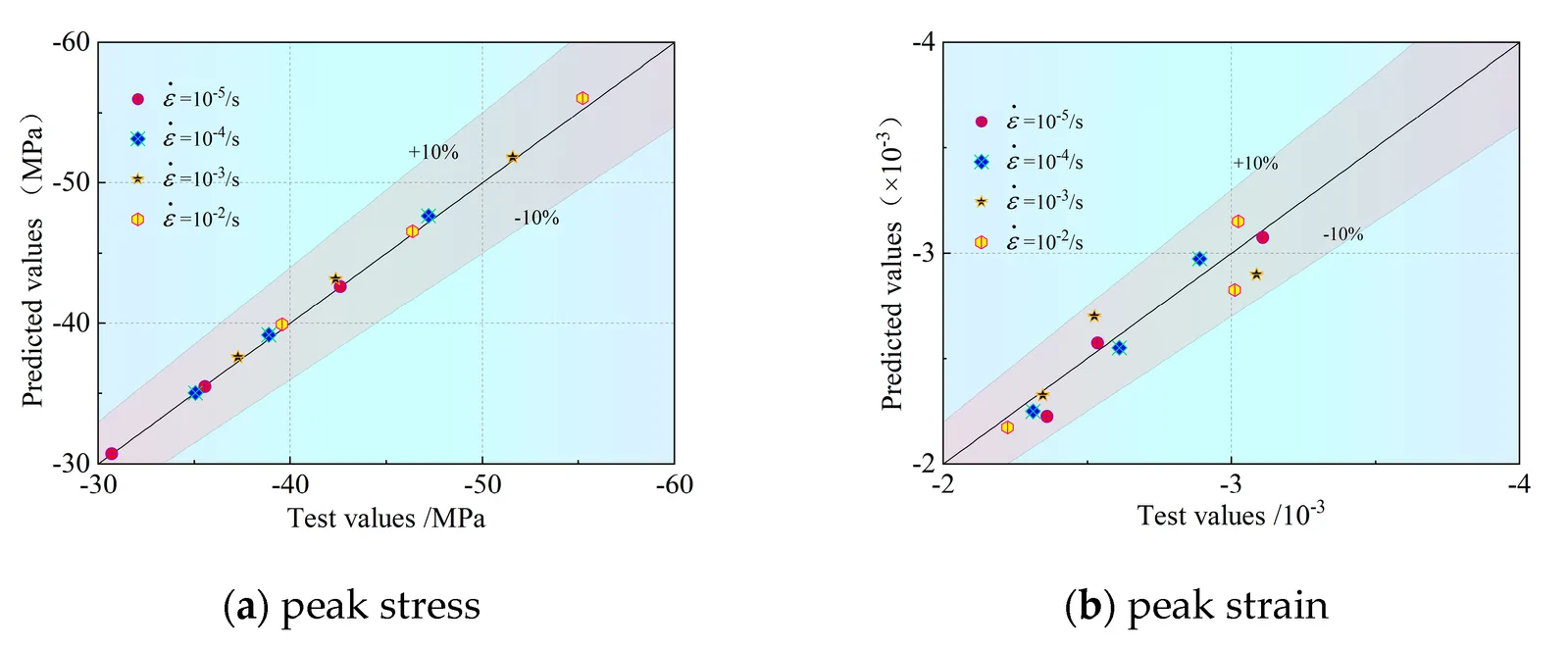
Plot of peak comparison of stress and strain.

Figure 12 presents both the experimental stress–strain curves and the model-predicted curves under different CF contents and strain rates. The predicted results are in good agreement with the experimental data. Figure 13 shows the effective stress–strain curves predicted by the damage model: during the homogeneous damage stage, the effective stress increases steadily and reaches its peak at the critical state, after which the specimen enters the local failure stage. The figure clearly indicates that with increasing CF content, the stress corresponding to the critical state gradually increases while the corresponding strain decreases. With increasing strain rate, the critical stress also gradually rises, whereas the strain does not exhibit a clear regular trend. As shown in Figure 14, the deviations between the predicted values of peak stress and peak strain and the experimental results are all less than 10%, further demonstrating the satisfactory predictive performance of the model.

**Figure 15:**
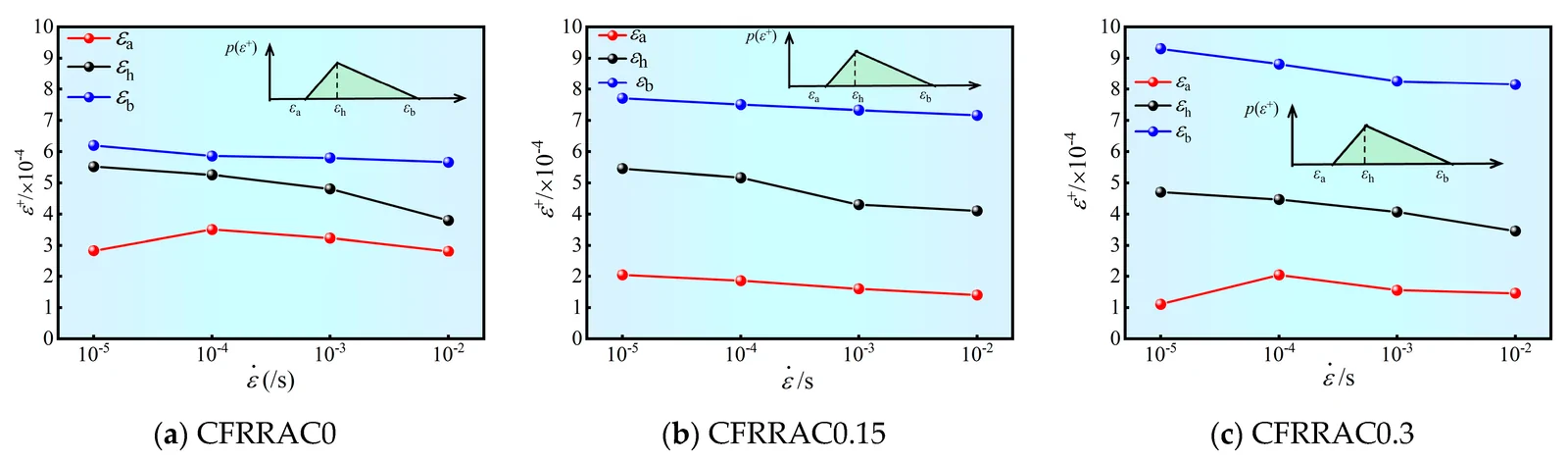
$\varepsilon^{+}$-$\dot{\varepsilon }$ curves.

Figure 15 illustrates the variations of the characteristic parameters εa, εh and εb with strain rate for specimens with three different CF contents. These three parameters jointly govern the triangular distribution shape of *p*(*ε*^+^), and their trends can reflect the influence of strain rate on the mesoscopic yielding damage evolution of concrete. Taking the CFRRAC0.15 specimens as an example, as $\dot{\varepsilon }$ increased from 10^−5^/s to 10^−2^/s, *ε*_a_ decreased from 2.044 × 10^−4^ to 1.406 × 10^−4^, *ε*_h_ decreased from 5.461 × 10^−4^ to 4.104 × 10^−4^, and *ε*_b_ decreased from 7.706 × 10^−4^ to 7.16 × 10^−4^; all three parameters decreased with increasing strain rate. The three characteristic parameters of the CFRRAC0 and CFRRAC0.3 specimens also exhibited a similar decreasing trend, indicating that an increase in strain rate accelerates the mesoscopic damage evolution process of specimens with different CF contents.

**Figure 16:**
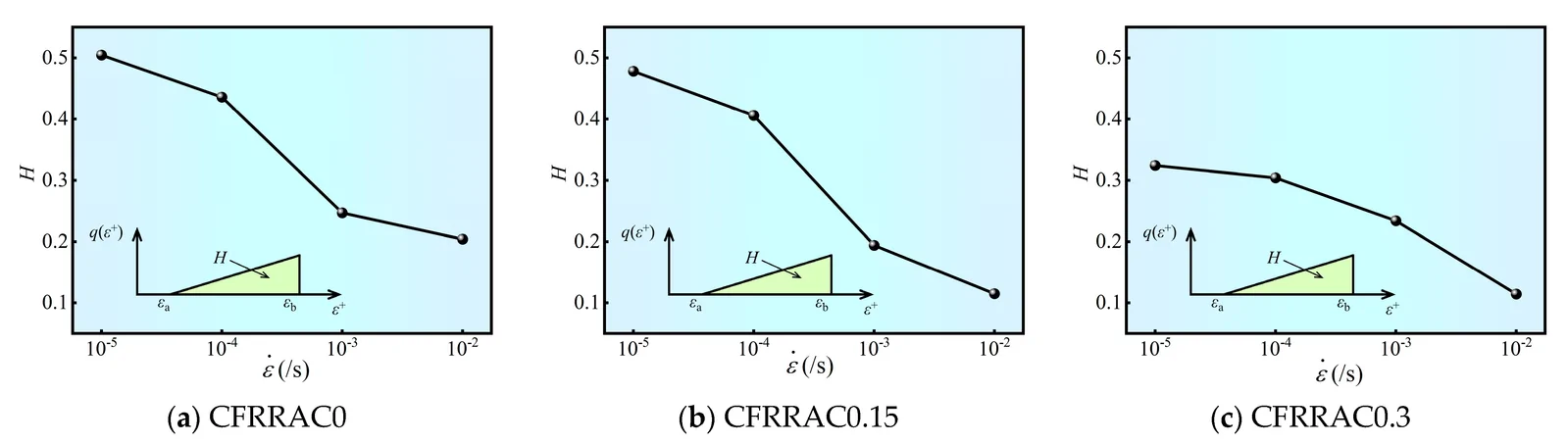
*H*-$\dot{\varepsilon }$ curves.

Figure 16 depicts the relationship between strain rate and the fracture damage-related parameter *H*. For all three groups of specimens, *H* decreases linearly with increasing strain rate. At the same strain rate, the *H* value decreases with increasing CF content. Taking the specimens at $\dot{\varepsilon }$ = 10^−5^/s as an example, the *H* values of CFRRAC0, CFRRAC0.15 and CFRRAC0.3 are 0.505, 0.478 and 0.324, respectively; this trend also holds for the other strain rates. For the same CF content, the H value also decreases with increasing strain rate. For instance, the H value of CFRRAC0.3 decreases from 0.324 at $\dot{\varepsilon }$ = 10^−5^/s to 0.114 at $\dot{\varepsilon }$ = 10^−2^/s representing a reduction of 64.8%; this pattern also applies to the CFRRAC0 and CFRRAC0.15 specimens. Since the degree of fracture damage is associated with microcrack density, the above trends imply that with increasing CF content and strain rate, the microcrack density within the specimens decreases, which is consistent with the experimental observations.

**Figure 17:**
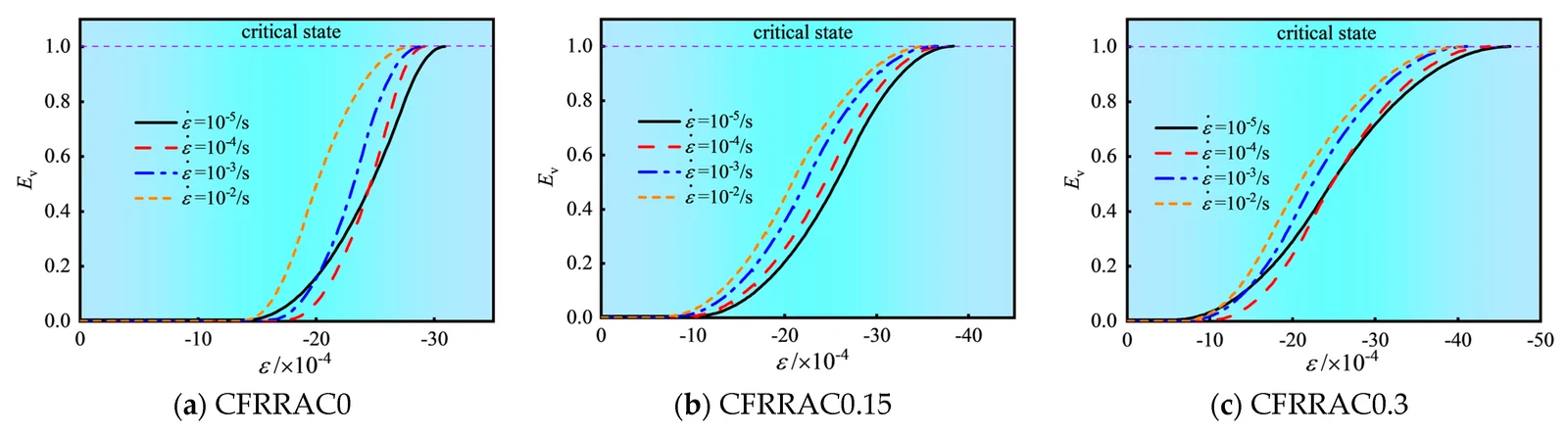
$E_{v}$ evolution curves.

Figure 17 presents the evolution curves of $E_{v}$ for the three groups of specimens. The evolution factor $E_{v}$ reflects the degree of adjustment of the microstructural load-bearing skeleton and plays a key role during the deformation process. During the homogeneous damage stage, its value ranges from 0 to 1, reflecting the transition of mesoscopic damage evolution from quantitative to qualitative change. Examining the curve morphology, it can be observed that increasing the strain rate tends to make the $E_{v}$ curves of all specimens’ steeper. However, the variation in the strain corresponding to initial damage exhibits a clear differentiation depending on the CF content: for the CFRRAC0.15 specimens, the initial damage strain gradually decreases with increasing strain rate, whereas for the CFRRAC0 and CFRRAC0.3 specimens, this strain does not show a consistent trend. In addition, the range from initial damage to the critical state for the CFRRAC0.15 and CFRRAC0.3 specimens is noticeably larger than that for the CFRRAC0 specimens, indicating that the incorporation of CF endows the material with enhanced deformation capacity and ductility under dynamic loading. When $E_{v}$ =1, the critical state is reached, implying that the microstructural load-bearing skeleton has been adjusted to its optimum and the latent mechanical performance has reached its limit, after which the specimen enters the local failure stage.

**Figure 18:**
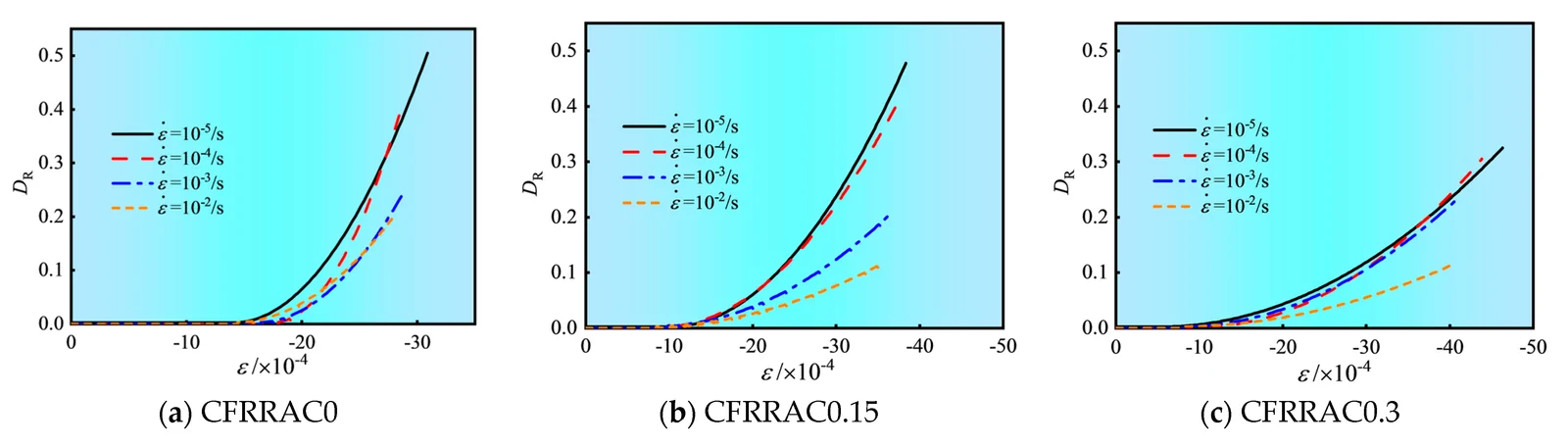
*D*_R_ evolution curves.

Figure 18 presents the evolution curves of the fracture damage variable *D*_R_ for the CFRRAC0, CFRRAC0.15 and CFRRAC0.3 specimens at different strain rates. The initiation and propagation of microcracks are closely related to *D*_R_. At $\dot{\varepsilon }$ = 10^−5^/s, the maximum values of *D*_R_ for the CFRRAC0, CFRRAC0.15 and CFRRAC0.3 specimens are 0.505, 0.478 and 0.324, respectively. At $\dot{\varepsilon }$ = 10^−2^/s, the maximum values of *D*_R_ decrease by 59.6%, 75.9% and 64.8%, respectively, relative to those at 10^−5^/s. The results indicate that with increasing strain rate, the evolution process of *D*_R_ for all specimens is completed within a smaller strain range. At high strain rates, the accumulation of internal damage in the specimens is more rapid, and the critical damage state can be reached under relatively small macroscopic deformation.

**Figure 19:**
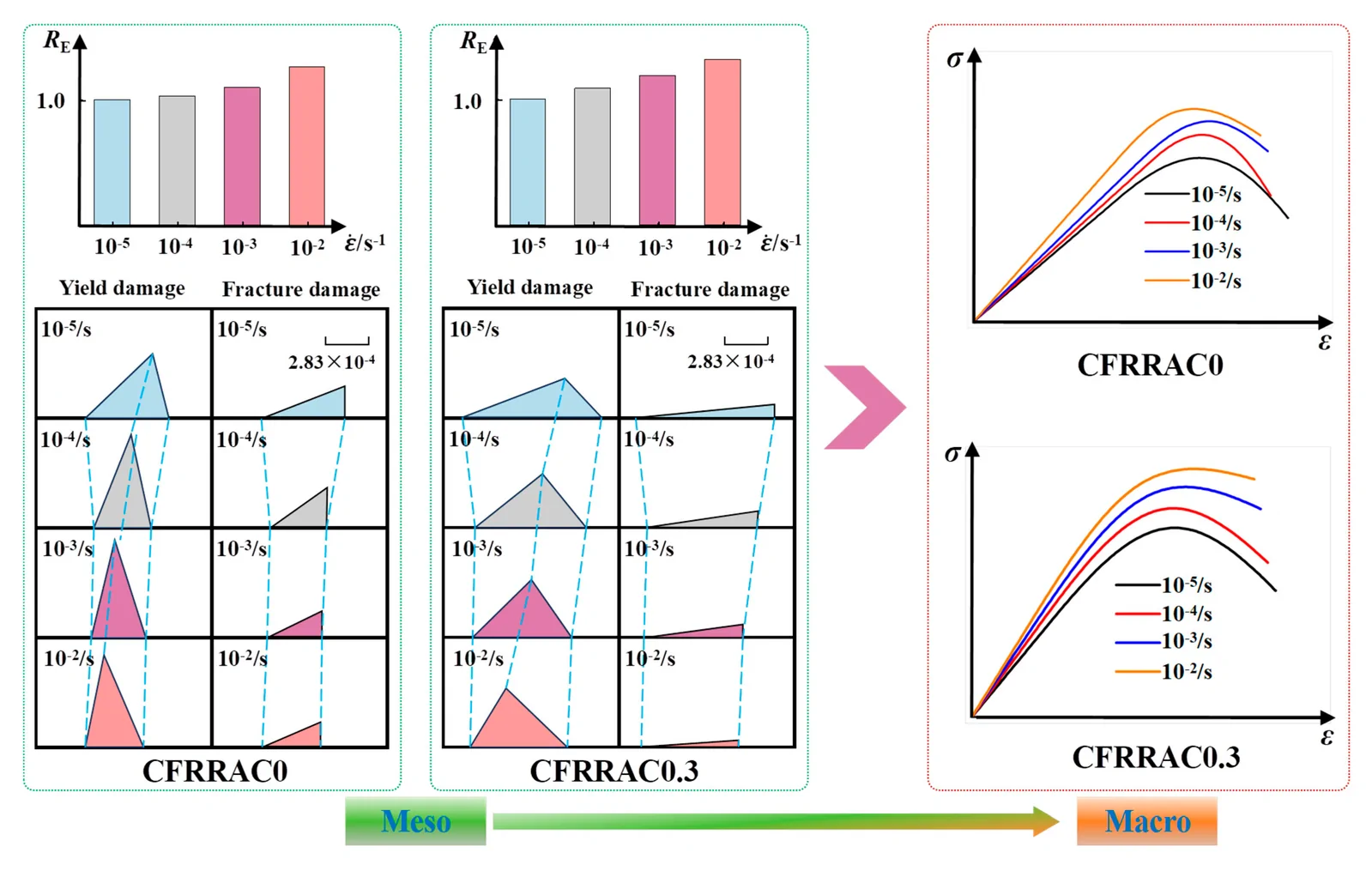
Relationship between meso-damage evolution and macro stress–strain.

Figure 19 illustrates the evolution of the characteristic parameters of the CFRRAC0, CFRRAC0.15 and CFRRAC0.3 specimens with dynamic strain rate. It can be observed from the figure that, as the strain rate increases, RE generally exhibits an upward trend. The functions *p*(*ε*^+^) and *q*(*ε*^+^) display a pronounced triangular distribution feature. With increasing strain rate, the apex of the triangle gradually shifts to the left (indicating a decrease in *ε*_h_), and the distribution interval tends to narrow (as reflected by the decreasing difference between *ε*_b_ and *ε*_a_). Notably, the narrowing of the interval for the CFRRAC0.3 specimens is less pronounced than that for the CFRRAC0 specimens, which is attributed to the bridging effect of CF (see Section 3.3). The above damage spectra intuitively reveal the mesoscopic damage evolution mechanisms of CFRRAC, which further influence the macroscopic nonlinear stress–strain behavior.

## 5. Conclusions

In this study, the effects of CF content (0%, 0.15% and 0.3%) and strain rate (10^−5^/s, 10^−4^/s, 10^−3^/s, 10^−2^/s) on the mechanical properties, microstructure, pore characteristics and mesoscopic damage evolution of CFRRAC were systematically investigated through uniaxial compression tests, SEM, AE and mesoscopic statistical damage theory. The main conclusions are summarized as follows:

(1) The mechanical properties of CFRRAC exhibit regular variations with CF content and strain rate. At the same strain rate, compared with the CFRRAC0 specimens, the peak stress of the CFRRAC0.3 specimens increased by 37.16–41.18% over the strain rate range of 10^−5^/s to 10^−2^/s, and the peak strain increased by 22.94–36.57%. For the same CF content, taking the CFRRAC0.3 specimens as an example, the peak stress at strain rates of 10^−4^/s, 10^−3^/s and 10^−2^/s increased by 9.31%, 18.66% and 31.24%, respectively, compared with that under quasi-static loading.
(2) At low strain rates, the loading rate is relatively low, and the specimens require a longer time to reach failure. Microcracks initiate from the ITZ and propagate progressively, resulting in a larger number of microcracks with smaller widths at failure. At high strain rates, the loading rate is high, and microcracks do not have sufficient time to fully propagate. They tend to cut through some of the aggregates, forming penetrating cracks with larger widths but fewer in number.
(3) CF enhances the mechanical performance of RAC by improving its microstructural characteristics. The failure modes of CFRRAC mainly include fiber pull-out and fracture. The uniform distribution of CF in the matrix and its bridging effect can effectively transfer and redistribute the internal tensile stresses in concrete, inhibit microcrack propagation, and thereby improve the mechanical properties of RAC. In addition, the AE peak region does not correspond to the peak stress point of the stress–strain curve, but rather lags it, and this position coincides exactly with the critical state in the statistical model.
(4) The macroscopic nonlinear stress–strain behavior is governed by the initial mechanical properties and the two damaged evolution processes of mesoscopic fracture and yielding. The damage evolution can be intuitively reflected by the damage spectra obtained in this study. With increasing CF content and strain rate, the mesoscopic damage characteristic parameters (*ε*_a_, *ε*_b_, *ε*_h_ and *H*) generally exhibit a decreasing trend, while the evolution curves of $E_{v}$ and *D*_R_ show clear regular variations. The damage spectra intuitively reveal the mesoscopic damage evolution mechanisms of CFRRAC, effectively establishing a link between these mechanisms and the macroscopic nonlinear stress–strain behavior.

The improved strain rate sensitivity, enhanced peak stress, and retained ductility of CFRRAC endow it with promising application potential in dynamic and impact-resistant engineering structures, including transportation infrastructures, industrial floors, and protective facilities. However, this study has certain limitations. The obtained results are validated only under laboratory-scale test conditions, and further verification via large-scale component tests and complex loading tests is still required for practical engineering promotion. In addition, this work only conducts a qualitative analysis of the relationship between AE parameters and energy dissipation. A detailed quantitative analysis of their internal correlation will be carried out in future studies to further clarify the damage evolution mechanism of CFRRAC.

## Figures and Tables

**Table 1:** Chemical composition of cement (wt.%).

CaO	SiO_2_	Al_2_O_3_	Fe_2_O_3_	SO_3_	Na_2_O	K_2_O	TiO_2_	MgO
53.49	23.88	9.54	2.73	2.68	0.951	0.777	0.579	4.49

**Table 2:** Performance index of CFs.

Carbon Content /%	Long /mm	Caliber/µm	Section Shape	Packing Density (g/cm^3^)	Densities (g/cm^3^)	Tensile Strength (MPa)	Tensile Modulus (GPa)
95	9	7	orbicular	0.4	1.75	4900	228

**Table 3:** Performance indexes of RCA.

Aggregate Size/mm	Performance Density (kg/m^3^)	Moisture Content/%	Water Absorption/%	Indicators of Crushing/%	Porosity/%
5–20	2680	3.02	5.89	12.39	51.00

**Table 4:** Mix proportions of specimens with different CF content (kg/m^3^).

Number	Cement	Sand	RCA	Water	Additional Water	CF	Water Reducing Agent	Dispersing Agent	Defoamer
CFRRAC0	360	646	1228	166	15.2	0	1.08	0	0
CFRRAC0.15						2.63		1.44	0.36
CFRRAC0.3						5.25		1.44	0.36

**Table 5:** Calculation parameters.

	$\dot{\varepsilon }$ (1/s)	$R_{E}$	*ε*_a_/10^−4^	*ε*_h_/10^−4^	*ε*_b_/10^−4^	*H*
CFRRAC0	10^−5^	1.000	2.830	5.513	6.203	0.505
	10^−4^	1.061	3.506	5.259	5.861	0.436
	10^−3^	1.135	3.327	4.808	5.802	0.247
	10^−2^	1.324	2.816	3.795	5.656	0.204
CFRRAC0.15	10^−5^	1.000	2.044	5.461	7.706	0.478
	10^−4^	1.126	1.854	5.161	7.506	0.406
	10^−3^	1.225	1.605	4.309	7.324	0.194
	10^−2^	1.336	1.406	4.104	7.160	0.115
CFRRAC0.3	10^−5^	1.000	1.107	4.705	9.306	0.324
	10^−4^	1.071	2.047	4.475	8.806	0.304
	10^−3^	1.257	1.547	4.075	8.256	0.234
	10^−2^	1.361	1.457	3.453	8.156	0.114

## Data Availability

The original contributions presented in this study are included in the article. Further inquiries can be directed to the corresponding authors.
